# Supplementary material for: Differential conformational dynamics in two type-A RNA-binding domains drive the double-stranded RNA recognition and binding
Source: eLife. 2024 Aug 8;13:RP94842. doi: 10.7554/eLife.94842 (PMC11309768; doi:10.7554/eLife.94842)
Supplement: Supplementary file 1. — (a) Amino acid length of different secondary structures in TRBP2-dsRBD1 and TRBP2-dsRBD2 CS-ROSSETA structures. α represents an α-helix, L represents a loop, and β represents the β-strand. (b) RNA sequences used to study interaction with dsRBDs. (c) Nuclear spin relaxation data for apo TRBP2-dsRBD2 recorded at 600 and 800 MHz NMR spectrometer. Data for some residues are missing in this table due to line-broadening issues in the corresponding experiments. (d) Order parameter (S2) extracted from model-free analysis of the nuclear spin relaxation data for apo TRBP2-dsRBD2 recorded at 600 and 800 MHz NMR spectrometer. (e) Rex of residues extracted from model-free analysis of the nuclear spin relaxation data for apo TRBP2-dsRBD2 recorded at 600 and 800 MHz NMR spectrometer. (f) R2,eff values measured at different Carr–Purcell–Meiboom–Gill (CPMG) frequencies from CPMG relaxation dispersion experiment for apo TRBP2-dsRBD2 at 600 MHz NMR spectrometer. (g) R1ρ relaxation rates measured using HSn pulses (n = 1, 2, 4, 6, 8) from heteronuclear adiabatic relaxation dispersion (HARD) experiment for apo TRBP2-dsRBD2 at 600 MHz NMR spectrometer. (h) R2ρ relaxation rates measured using HSn pulses (n = 1, 2, 4, 6, 8) from HARD experiment for apo TRBP2-dsRBD2 at 600 MHz NMR spectrometer. (i) Dynamics parameters extracted from HARD experimental data from geoHARD method for apo TRBP2-dsRBD2. (j) Isothermal titration calorimetry (ITC)-binding study of TRBP2-dsRBD2 and D12 RNA carried out in triplicate. (k) Nuclear spin relaxation data for RNA-bound TRBP2-dsRBD2 recorded at 600 and 800 MHz NMR spectrometer. Data for some residues are missing in this table due to line-broadening issues in the corresponding experiments. (l) Order parameter (S2) extracted from model-free analysis of the nuclear spin relaxation data for RNA-bound TRBP2-dsRBD2 recorded at 600 and 800 MHz NMR spectrometer. (m) Rex of residues extracted from model-free analysis of nuclear spin relaxation data for RNA-bound TRBP2 [file elife-94842-supp1.docx]

**Supplementary File 1**

**Supplementary File 1a**: Amino acid length of different secondary structures in TRBP2-dsRBD1 and TRBP2-dsRBD2 CS-ROSSETA structures. α represents an α-helix, L represents a loop, and β represents the β-strand.

| Domain Construct | α1 | L1 | β1 | L2 | β2 | L3 | β3 | L4 | α2 |
| --- | --- | --- | --- | --- | --- | --- | --- | --- | --- |
| TRBP2-dsRBD1 | 10 | 4 | 8 | 5 | 9 | 2 | 7 | 2 | 17 |
| TRBP2-dsRBD2 | 10 | 5 | 8 | 6 | 8 | 4 | 6 | 2 | 15 |

**Supplementary File 1b**: RNA sequences used to study interaction with dsRBDs.

| **Name** | **Strand** | **Sequence (5ʹ 🡪 3ʹ)** |
| --- | --- | --- |
| **miR-16-1-A** | Guide | UAGCAGCACGUAAAUAUUGGCG |
|  | Passenger | CCAGUAUUAACUGUGCUGCUGAA |
| **miR-16-1-D** | Guide | UAGCAGCACGUAAAUAUUGGCG |
|  | Passenger | CCAGUAUUUACGUGCUGCUGAA |
| **miR-16-1-M** | Guide | UAGCAGCACGUAAAUAUUGGCG |
|  | Passenger | CCAGUAUUAACGUGCUGCUGAA |
| **miR-16-1-B** | Guide | UAGCAGCACGUAAAUAUUGGCG |
|  | Passenger | CCAGUAUUAACGUGCUGCUGAA |
| **D12 RNA** | Guide | CGUAAAUAUUCG |
|  | Passenger | CGAGUAUUUACG |

**Supplementary File 1c**: Nuclear spin relaxation data for apo TRBP2-dsRBD2 recorded at 600 MHz and 800 MHz NMR spectrometer. Data for some residues is missing in this table due to line-broadening issues in the corresponding experiments.

| **Residue Number** | **600 MHz** | | | | | | **800 MHz** | | | | | |
| --- | --- | --- | --- | --- | --- | --- | --- | --- | --- | --- | --- | --- |
|  | ***R_1_* (s^-1^)** | | ***R_2_* (s^-1^)** | | **NOE** | | ***R1* (s^-1^)** | | ***R_2_* (s^-1^)** | | **NOE** | |
|  | **Value** | **Error** | **Value** | **Error** | **Value** | **Error** | **Value** | **Error** | **Value** | **Error** | **Value** | **Error** |
| S151 | 1.39 | 0.04 | 3.32 | 0.05 | -0.31 | 0.01 | 1.34 | 0.04 | 4.98 | 1.40 | -0.07 | 0.01 |
| N152 | 1.41 | 0.07 | 4.23 | 0.09 | -13 | 0.01 | 1.41 | 0.05 | 5.67 | 1.41 | 0.38 | 0.01 |
| A153 | 1.38 | 0.07 | 5.36 | 0.08 | 0.12 | 0.01 | 1.42 | 0.02 | 5.91 | 0.33 | 0.38 | 0.01 |
| Q154 | 1.45 | 0.05 | 4.02 | 0.05 | 0.09 | 0.01 | 1.48 | 0.04 | 4.73 | 0.62 | 0.40 | 0.01 |
| Q155 | 1.33 | 0.05 | 3.60 | 0.1 | 0.14 | 0.01 | 1.37 | 0.03 | 4.5 | 0.89 | 0.77 | 0.01 |
| S156 | 1.45 | 0.06 | 4.49 | 0.06 | -0.05 | 0.01 | 1.46 | 0.03 | 6.24 | 1.42 | 0.25 | 0.01 |
| S157 | 1.51 | 0.06 | 5.22 | 0.13 | 0.23 | 0.01 | 1.51 | 0.05 | 6.57 | 1.27 | 0.39 | 0.01 |
| N159 | 1.38 | 0.04 | 7.64 | 0.18 | 0.48 | 0.03 | 1.26 | 0.06 | 8.61 | 1.59 | 0.61 | 0.03 |
| G162 | 1.45 | 0.02 | 10.86 | 0.14 | 0.78 | 0.02 | 1.20 | 0.03 | 10.20 | 0.74 | 0.84 | 0.02 |
| A163 | 1.32 | 0.04 | 11.55 | 0.28 | 0.81 | 0.04 | 1.22 | 0.02 | 12.27 | 0.47 | 0.82 | 0.02 |
| L164 | 1.40 | 0.02 | 12.54 | 0.19 | 0.83 | 0.02 | 1.11 | 0.03 | 12.25 | 0.38 | 0.85 | 0.02 |
| Q165 | 1.28 | 0.06 | 12.66 | 0.11 | 0.82 | 0.02 | 1.24 | 0.01 | 12.03 | 0.46 | 0.84 | 0.01 |
| E166 | 1.43 | 0.02 | 11.88 | 0.11 | 0.81 | 0.01 | 1.12 | 0.02 | 11.40 | 0.24 | 0.84 | 0.01 |
| L167 | 1.35 | 0.06 | 12.41 | 0.45 | 0.76 | 0.04 | 1.12 | 0.01 | 12.18 | 0.11 | 0.79 | 0.02 |
| V168 | 1.37 | 0.06 | 21.02 | 1.17 | 0.92 | 0.06 | 1.10 | 0.03 | - | - | 0.88 | 0.04 |
| V169 | 1.46 | 0.09 | 10.85 | 0.73 | 0.69 | 0.04 | 1.17 | 0.02 | 11.68 | 0.31 | 0.87 | 0.04 |
| Q170 | 1.45 | 0.02 | 12.11 | 0.25 | 0.80 | 0.01 | 1.10 | 0.02 | 11.69 | 0.10 | 0.82 | 0.01 |
| G172 | 1.44 | 0.05 | 10.56 | 0.89 | 0.76 | 0.02 | 1.21 | 0.01 | 10.80 | 0.32 | 0.81 | 0.02 |
| W173 | 1.29 | 0.10 | 10.44 | 0.30 | 0.80 | 0.03 | 1.22 | 0.01 | 10.15 | 0.16 | 0.84 | 0.02 |
| R174 | 1.36 | 0.03 | 9.48 | 0.32 | 0.8 | 0.05 | 1.11 | 0.03 | 10.16 | 0.13 | 0.78 | 0.03 |
| L175 | 1.46 | 0.04 | 10.70 | 0.1 | 0.80 | 0.02 | 1.18 | 0.02 | 10.09 | 0.13 | 0.80 | 0.02 |
| E177 | 1.39 | 0.02 | 4.99 | 0.39 | 0.57 | 0.01 | 1.15 | 0.04 | 9.65 | 0.56 | 0.59 | 0.01 |
| Y178 | 1.45 | 0.07 | 9.60 | 0.66 | 0.74 | 0.03 | 1.32 | 0.02 | 10.13 | 0.60 | 0.80 | 0.02 |
| T179 | 1.50 | 0.03 | 10.54 | 00.12 | 0.72 | 0.02 | 1.25 | 0.03 | 10.69 | 0.69 | 0.80 | 0.02 |
| V180 | 1.54 | 0.09 | 9.89 | 1.48 | 0.68 | 0.04 | 1.42 | 0.05 | 12.93 | 1.34 | 0.88 | 0.05 |
| T181 | 1.56 | 0.02 | 10.22 | 0.31 | 0.78 | 0.02 | 1.28 | 0.04 | 10.42 | 0.64 | 0.85 | 0.02 |
| Q182 | 1.54 | 0.03 | 11.54 | 0.37 | 0.71 | 0.02 | 1.32 | 0.04 | 12.09 | 0.85 | 0.79 | 0.02 |
| E183 | 1.43 | 0.03 | 10.61 | 0.08 | 0.65 | 0.01 | 1.28 | 0.04 | 10.30 | 0.76 | 0.70 | 0.01 |
| S184 | 1.41 | 0.04 | - | - | 0.52 | 0.05 | 1.35 | 0.03 | - | - | 0.65 | 0.04 |
| G185 | 1.44 | 0.03 | 8.30 | 0.18 | 0.57 | 0.02 | 1.29 | 0.05 | 8.71 | 1.11 | 0.64 | 0.02 |
| R189 | 1.58 | 0.05 | 15.44 | 0.69 | 0.49 | 0.05 | 1.40 | 0.06 | 16.65 | 1.49 | 0.51 | 0.04 |
| E191 | 1.41 | 0.02 | 9.07 | 0.06 | 0.54 | 0.01 | 1.18 | 0.03 | 9.23 | 0.49 | 0.60 | 0.01 |
| F192 | 1.40 | 0.03 | 12.40 | 0.29 | - | - | 1.22 | 0.03 | 12.16 | 0.53 | 0.79 | 0.02 |
| T193 | 1.42 | 0.02 | 12.39 | 0.14 | 0.78 | 0.03 | 1.13 | 0.03 | 11.67 | 0.67 | 0.84 | 0.02 |
| M194 | 1.52 | 0.08 | 9.92 | 0.56 | 0.72 | 0.07 | 1.31 | 0.04 | 10.71 | 0.50 | 0.80 | 0.06 |
| T195 | 1.51 | 0.03 | 13.36 | 0.15 | 0.78 | 0.03 | 1.18 | 0.04 | 13.47 | 0.70 | 0.85 | 0.02 |
| C196 | 1.56 | 0.03 | 11.00 | 0.28 | 0.82 | 0.04 | 1.23 | 0.04 | 10.85 | 0.52 | 0.78 | 0.02 |
| R197 | 1.51 | 0.03 | 8.40 | 0.94 | 0.84 | 0.05 | 1.22 | 0.04 | 9.64 | 0.50 | 0.81 | 0.04 |
| V198 | 1.54 | 0.11 | 9.52 | 0.64 | 0.73 | 0.04 | 1.20 | 0.03 | 10.49 | 0.61 | 0.82 | 0.05 |
| E199 | 1.39 | 0.09 | 10.27 | 0.15 | 0.68 | 0.02 | 1.22 | 0.02 | 9.77 | 0.29 | 0.76 | 0.01 |
| R200 | 1.31 | 0.07 | 11.83 | 0.57 | 0.63 | 0.05 | 1.07 | 0.04 | 10.69 | 0.38 | 0.82 | 0.05 |
| F201 | 1.59 | 0.04 | 10.67 | 0.13 | 0.72 | 0.01 | 1.27 | 0.03 | 10.73 | 0.38 | 0.79 | 0.02 |
| I202 | 1.55 | 0.04 | 9.82 | 0.21 | 0.74 | 0.02 | 1.19 | 0.03 | 9.86 | 0.41 | 0.86 | 0.02 |
| E203 | 1.62 | 0.08 | 10.21 | 0.13 | 0.77 | 0.01 | 1.29 | 0.02 | 9.83 | 0.44 | 0.82 | 0.02 |
| I204 | 1.33 | 0.05 | 9.87 | 0.22 | 0.68 | 0.04 | 1.11 | 0.04 | 9.54 | 0.33 | 0.72 | 0.03 |
| G205 | 1.56 | 0.03 | 10.33 | 0.10 | 0.77 | 0.02 | 1.22 | 0.04 | 10.27 | 0.39 | 0.83 | 0.02 |
| S206 | 1.50 | 0.02 | 10.37 | 0.13 | 0.72 | 0.01 | 1.16 | 0.03 | 10.55 | 0.43 | 0.80 | 0.02 |
| G207 | 1.55 | 0.07 | 10.48 | 0.66 | 0.79 | 0.01 | 1.32 | 0.01 | 11.35 | 0.72 | 0.80 | 0.02 |
| T208 | 1.54 | 0.10 | 10.31 | 0.14 | 0.75 | 0.02 | 1.37 | 0.04 | 10.68 | 1.25 | 0.83 | 0.02 |
| S209 | 1.29 | 0.09 | 6.42 | 0.69 | - | - | - | - | - | - | - | - |
| K210 | 1.60 | 0.14 | 8.69 | 0.7 | 0.75 | 0.06 | 1.30 | 0.06 | 9.23 | 1.02 | 0.85 | 0.12 |
| L212 | 1.28 | 0.05 | 12.31 | 0.44 | 0.72 | 0.04 | 1.15 | 0.04 | 12.44 | 0.88 | 0.81 | 0.02 |
| A213 | 1.50 | 0.05 | 12.52 | 0.35 | 0.88 | 0.03 | 1.22 | 0.03 | 12.33 | 0.59 | 0.82 | 0.03 |
| R215 | 1.27 | 0.04 | 12.93 | 0.41 | 0.66 | 0.06 | 1.10 | 0.02 | 12.33 | 0.34 | 0.81 | 0.03 |
| N216 | 1.37 | 0.04 | 12.83 | 0.15 | 0.83 | 0.01 | 1.13 | 0.02 | 12.74 | 0.44 | 0.83 | 0.01 |
| A217 | 1.48 | 0.02 | 11.11 | 0.36 | - | - | 1.19 | 0.03 | 12.01 | 0.73 | 0.88 | 0.03 |
| A218 | 1.45 | 0.04 | 12.37 | 0.40 | 0.79 | 0.02 | 1.19 | 0.02 | 12.47 | 0.40 | 0.81 | 0.04 |
| A219 | 1.44 | 0.04 | 12.61 | 0.18 | 0.84 | 0.02 | 1.13 | 0.03 | 12.36 | 0.31 | 0.88 | 0.03 |
| M221 | 1.46 | 0.03 | 8.57 | 0.22 | 0.5 | 0.02 | 1.25 | 0.06 | 9.11 | 1.29 | 0.49 | 0.02 |
| L222 | 1.56 | 0.02 | - | - | 0.79 | 0.03 | 1.13 | 0.02 | 12.70 | 0.26 | 0.86 | 0.02 |
| L223 | 1.31 | 0.03 | 12.49 | 0.53 | 0.65 | 0.03 | 1.07 | 0.01 | 12.44 | 0.24 | 0.87 | 0.02 |
| R224 | 1.20 | 0.06 | 11.11 | 0.90 | 0.79 | 0.05 | 1.15 | 0.03 | 11.33 | 0.36 | 0.84 | 0.03 |
| V225 | 1.30 | 0.09 | 12.12 | 0.32 | 0.76 | 0.05 | 1.16 | 0.01 | 11.29 | 0.38 | 0.79 | 0.04 |
| T227 | 1.50 | 0.04 | 8.77 | 0.16 | 0.54 | 0.02 | 1.39 | 0.04 | 9.60 | 1.33 | 0.66 | 0.02 |
| V228 | 1.57 | 0.04 | 7.61 | 0.19 | 0.57 | 0.02 | 1.36 | 0.05 | 7.88 | 0.59 | 0.64 | 0.02 |
| L230 | 1.01 | 0.08 | 1.62 | 0.41 | -0.65 | 0.00 | 0.95 | 0.02 | 1.97 | 0.37 | -0.81 | 0.01 |
| A232 | 1.44 | 0.05 | 4.08 | 0.06 | -0.36 | -0.02 | 1.31 | 0.04 | 4.54 | 0.61 | 0.29 | 0.02 |

**Supplementary File 1d**: Order parameter (S^2^) extracted from Model-free analysis of the Nuclear Spin Relaxation data for apo TRBP2-dsRBD2 recorded at 600 MHz and 800 MHz NMR spectrometer.

| **Residue Number** | **S^2^** | |
| --- | --- | --- |
|  | **Value** | **Error** |
| G162 | 0.78 | 0.01 |
| A163 | 0.40 | 0.01 |
| L164 | 0.85 | 0.01 |
| Q165 | 0.80 | 0.00 |
| E166 | 0.81 | 0.01 |
| L167 | 0.73 | 0.01 |
| V169 | 0.62 | 0.02 |
| Q170 | 0.75 | 0.00 |
| G172 | 0.63 | 0.03 |
| W173 | 0.63 | 0.02 |
| R174 | 0.23 | 0.01 |
| L175 | 0.38 | 0.04 |
| E177 | 0.00 | 0.11 |
| Y178 | 0.60 | 0.04 |
| T179 | 0.75 | 0.01 |
| V180 | 0.12 | 0.28 |
| T181 | 0.73 | 0.03 |
| Q182 | 0.56 | 0.02 |
| E183 | 0.31 | 0.01 |
| G185 | 0.18 | 0.01 |
| R189 | 0.52 | 0.03 |
| E191 | 0.44 | 0.01 |
| T193 | 0.88 | 0.01 |
| M194 | 0.68 | 0.03 |
| T195 | 0.73 | 0.01 |
| C196 | 0.82 | 0.01 |
| R197 | 0.50 | 0.05 |
| V198 | 0.34 | 0.04 |
| R200 | 0.45 | 0.02 |
| F201 | 0.77 | 0.01 |
| I202 | 0.64 | 0.02 |
| E203 | 0.72 | 0.02 |
| I204 | 0.67 | 0.02 |
| G205 | 0.71 | 0.01 |
| S206 | 0.75 | 0.01 |
| G207 | 0.59 | 0.05 |
| T208 | 0.77 | 0.02 |
| K210 | 0.52 | 0.05 |
| L212 | 0.71 | 0.12 |
| A213 | 0.83 | 0.01 |
| R215 | 0.69 | 0.01 |
| N216 | 0.74 | 0.01 |
| A218 | 0.74 | 0.01 |
| A219 | 0.91 | 0.01 |
| M221 | 0.47 | 0.02 |
| L223 | 0.65 | 0.09 |
| R224 | 0.58 | 0.02 |
| V225 | 0.67 | 0.02 |
| T227 | 0.24 | 0.01 |

**Supplementary File 1e**: *R_ex_* of residues extracted from Model-free analysis of the Nuclear Spin Relaxation data for apo TRBP2-dsRBD2 recorded at 600 MHz and 800 MHz NMR spectrometer.

| **Residue Number** | ***R_ex_* (s^-1^)** | |
| --- | --- | --- |
|  | **Value** | **Error** |
| E166 | 0.42 | 0.12 |
| E177 | 2.62 | 0.78 |
| V180 | 4.85 | 2.06 |
| T195 | 2.4 | 0.22 |
| L212 | 1.22 | 1.17 |
| N216 | 1.14 | 0.19 |
| L223 | 0.87 | 0.73 |

**Supplementary File 1f**: *R_2_eff* values measured at different CPMG frequencies from CPMG relaxation dispersion experiment for apo TRBP2-dsRBD2 at 600 MHz NMR spectrometer.

| **Residue Number** | ***R_2eff_* at CPMG frequency (s^-1^)** | | | | | | | | | | | | | |
| --- | --- | --- | --- | --- | --- | --- | --- | --- | --- | --- | --- | --- | --- | --- |
|  |  | | | | | | | | | | | **Repeat** | | |
|  | **25** | **50** | **75** | **125** | **175** | **275** | **375** | **525** | **675** | **825** | **1000** | **125** | **375** | **825** |
| error | 0.52 | 0.48 | 0.43 | 0.44 | 0.33 | 0.33 | 0.30 | 0.30 | 0.25 | 0.25 | 0.28 | 0.38 | 0.28 | 0.28 |
| S151 | 2.95 | 5.77 | 1.83 | 5.00 | 6.54 | 4.92 | 4.35 | 6.83 | 6.54 | 7.41 | 8.30 | 4.55 | 7.14 | 7.45 |
| N152 | 1.00 | 1.58 | 1.58 | 2.70 | 1.58 | 3.48 | 4.64 | 5.68 | 6.02 | 6.59 | 6.41 | 2.64 | 2.75 | 6.75 |
| A153 | 2.84 | 3.59 | 3.67 | 3.32 | 3.83 | 4.44 | 4.50 | 4.44 | 5.03 | 5.33 | 8.28 | 3.25 | 4.97 | 5.36 |
| Q154 | 6.27 | 6.07 | 5.86 | 6.07 | 6.76 | 6.34 | 6.63 | 7.11 | 7.38 | 7.58 | 4.76 | 5.97 | 6.33 | 7.59 |
| Q155 | 2.47 | 1.93 | 2.26 | 2.56 | 2.74 | 2.79 | 3.21 | 3.27 | 4.02 | 4.46 | 5.62 | 3.07 | 2.79 | 4.51 |
| E157 | 2.47 | 2.30 | 2.41 | 2.21 | 2.07 | 4.02 | 4.26 | 4.10 | 7.20 | 5.18 | 4.97 | 2.34 | 3.93 | 5.25 |
| C158 | 1.79 | 2.10 | 3.75 | 5.53 | 4.45 | 5.53 | 6.20 | 4.11 | 5.29 | 7.71 | 8.22 | 3.24 | 3.91 | 8.05 |
| N159 | 12.80 | 14.90 | 15.90 | 3.78 | 8.93 | 7.97 | 6.66 | 12.30 | 12.60 | 14.00 | 10.70 | 6.62 | 7.44 | 14.20 |
| L167 | 9.37 | 9.58 | 9.83 | 9.79 | 9.98 | 10.80 | 11.40 | 11.70 | 11.60 | 13.30 | 13.80 | 9.82 | 11.10 | 13.40 |
| V168 | 15.40 | 15.40 | 15.90 | 16.00 | 15.70 | 16.70 | 17.50 | 17.50 | 16.50 | 18.00 | 18.50 | 15.50 | 17.80 | 17.30 |
| V169 | 8.40 | 9.12 | 8.68 | 9.27 | 8.55 | 9.03 | 9.91 | 10.00 | 10.50 | 10.70 | 11.60 | 8.85 | 9.84 | 10.70 |
| Q170 | 8.42 | 8.27 | 8.42 | 9.89 | 9.64 | 8.62 | 9.11 | 8.90 | 9.31 | 9.95 | 11.00 | 9.33 | 8.70 | 10.70 |
| G172 | 8.20 | 8.31 | 8.22 | 7.95 | 8.52 | 8.64 | 8.97 | 9.56 | 10.20 | 10.30 | 11.30 | 8.04 | 9.18 | 10.80 |
| W173 | 7.12 | 8.34 | 7.89 | 7.02 | 7.31 | 8.09 | 8.59 | 9.11 | 9.34 | 10.00 | 10.90 | 7.42 | 8.84 | 10.10 |
| L175 | 7.80 | 7.73 | 8.49 | 7.88 | 8.26 | 8.15 | 9.51 | 9.51 | 10.30 | 10.40 | 10.70 | 8.20 | 8.70 | 10.50 |
| E177 | 8.14 | 7.62 | 7.66 | 8.44 | 7.80 | 8.46 | 9.07 | 9.34 | 9.39 | 10.70 | 11.70 | 8.58 | 8.60 | 10.80 |
| Y178 | 7.78 | 6.44 | 7.96 | 7.64 | 7.72 | 8.28 | 7.62 | 84.20 | 8.77 | 11.50 | 11.20 | 8.04 | 9.44 | 11.20 |
| T179 | 8.50 | 7.54 | 8.68 | 8.93 | 9.16 | 9.30 | 9.41 | 9.64 | 11.20 | 11.00 | 11.10 | 8.91 | 8.63 | 10.90 |
| V180 | 11.20 | 10.60 | 10.10 | 9.96 | 8.91 | 9.77 | 10.10 | 7.47 | 11.20 | 11.20 | 14.40 | 9.25 | 9.55 | 11.30 |
| T181 | 8.24 | 8.59 | 9.01 | 7.14 | 8.89 | 8.04 | 8.63 | 9.86 | 9.81 | 9.64 | 10.30 | 7.46 | 8.48 | 9.27 |
| Q182 | 8.16 | 7.68 | 8.23 | 8.21 | 8.27 | 7.83 | 9.46 | 9.08 | 10.20 | 9.32 | 11.00 | 8.00 | 8.86 | 10.30 |
| E183 | 7.30 | 7.43 | 7.30 | 7.44 | 7.49 | 7.74 | 7.89 | 8.82 | 9.20 | 9.42 | 9.78 | 7.18 | 7.80 | 9.49 |
| G185 | 6.24 | 4.69 | 5.88 | 6.62 | 4.94 | 6.76 | 7.73 | 6.29 | 6.47 | 7.35 | 7.69 | 5.37 | 5.90 | 7.49 |
| A187 | 10.40 | 8.74 | 9.48 | 10.20 | 9.64 | 10.00 | 11.70 | 10.70 | 12.00 | 10.50 | 11.70 | 8.94 | 10.60 | 11.20 |
| R189 | 7.06 | 8.56 | 7.36 | 7.14 | 9.54 | 9.29 | 9.45 | 9.41 | 11.30 | 9.73 | 12.90 | 9.44 | 11.70 | 8.70 |
| E191 | 6.67 | 6.48 | 6.26 | 6.47 | 7.39 | 6.75 | 7.04 | 7.52 | 7.78 | 7.98 | 8.69 | 6.38 | 6.74 | 7.99 |
| F192 | 9.20 | 8.03 | 8.28 | 9.72 | 8.96 | 9.93 | 10.20 | 10.00 | 9.87 | 10.90 | 10.80 | 9.05 | 9.29 | 11.30 |
| T193 | 8.84 | 9.17 | 9.26 | 9.33 | 9.55 | 10.50 | 10.70 | 11.70 | 11.20 | 12.10 | 13.10 | 9.62 | 10.40 | 12.10 |
| M194 | 9.13 | 10.50 | 8.68 | 9.42 | 8.96 | 9.68 | 10.80 | 10.40 | 10.30 | 12.10 | 12.30 | 7.61 | 10.10 | 12.20 |
| T195 | 9.39 | 9.39 | 9.06 | 10.30 | 9.57 | 9.66 | 9.94 | 10.80 | 10.30 | 11.50 | 11.60 | 9.90 | 10.90 | 11.60 |
| C196 | 8.38 | 8.57 | 9.57 | 8.55 | 8.33 | 9.19 | 9.86 | 9.08 | 8.63 | 9.77 | 11.10 | 9.33 | 7.91 | 9.86 |
| R197 | 8.81 | 8.49 | 7.95 | 7.72 | 8.00 | 8.13 | 8.61 | 10.30 | 10.30 | 12.10 | 10.90 | 7.54 | 8.15 | 12.40 |
| V198 | 8.49 | 6.46 | 8.07 | 7.74 | 7.01 | 8.63 | 8.56 | 9.51 | 7.88 | 10.10 | 9.46 | 8.57 | 9.46 | 10.10 |
| E199 | 7.56 | 7.65 | 7.70 | 8.07 | 8.07 | 8.54 | 8.86 | 9.56 | 10.30 | 11.70 | 10.90 | 8.26 | 9.45 | 11.70 |
| R200 | 8.18 | 9.23 | 8.39 | 8.31 | 9.41 | 9.50 | 9.71 | 10.90 | 10.90 | 11.40 | 9.85 | 8.59 | 10.60 | 12.40 |
| F201 | 8.27 | 8.35 | 8.87 | 8.58 | 8.67 | 9.06 | 8.87 | 9.82 | 10.30 | 11.10 | 10.90 | 8.49 | 8.94 | 11.20 |
| I202 | 7.81 | 8.12 | 7.34 | 7.02 | 11.00 | 7.51 | 8.35 | 9.49 | 9.50 | 10.10 | 11.00 | 7.61 | 9.28 | 10.20 |
| E203 | 7.90 | 8.48 | 6.33 | 8.48 | 8.35 | 9.09 | 8.56 | 9.52 | 10.10 | 10.40 | 11.40 | 8.69 | 8.45 | 10.50 |
| I204 | 9.02 | 9.29 | 8.84 | 9.87 | 9.76 | 10.90 | 10.10 | 11.30 | 10.30 | 12.40 | 12.30 | 9.58 | 11.40 | 12.40 |
| G205 | 8.43 | 8.20 | 8.01 | 8.21 | 8.36 | 9.58 | 9.18 | 9.32 | 9.99 | 10.90 | 11.30 | 8.41 | 9.27 | 11.00 |
| S206 | 8.84 | 9.17 | 9.26 | 9.33 | 9.55 | 10.30 | 10.70 | 11.70 | 11.20 | 12.10 | 12.90 | 9.62 | 10.40 | 12.60 |
| G207 | 8.30 | 7.89 | 8.00 | 8.60 | 8.09 | 9.37 | 9.90 | 9.92 | 10.40 | 11.50 | 11.90 | 8.80 | 9.72 | 11.50 |
| T208 | 9.89 | 5.44 | 7.69 | 8.33 | 6.76 | 9.71 | 7.99 | 10.60 | 9.83 | 10.10 | 11.60 | 8.63 | 10.20 | 10.20 |
| K210 | 6.15 | 7.06 | 7.04 | 11.10 | 2.35 | 6.02 | 5.54 | 12.20 | 6.76 | 10.60 | 5.55 | 10.40 | 4.12 | 11.90 |
| L212 | 9.36 | 9.27 | 8.34 | 9.36 | 10.10 | 10.10 | 10.10 | 9.88 | 11.50 | 11.90 | 13.10 | 8.94 | 10.10 | 11.80 |
| A213 | 9.71 | 9.31 | 9.59 | 9.19 | 10.10 | 10.40 | 9.98 | 11.90 | 11.80 | 12.40 | 14.00 | 9.57 | 10.70 | 12.50 |
| R215 | 10.40 | 9.84 | 9.99 | 9.94 | 10.60 | 10.40 | 10.90 | 10.30 | 11.90 | 11.50 | 12.40 | 10.00 | 9.22 | 11.40 |
| N216 | 9.56 | 9.68 | 10.40 | 10.40 | 9.96 | 10.70 | 11.10 | 10.90 | 11.10 | 12.00 | 12.90 | 9.93 | 10.80 | 12.10 |
| A217 | 8.19 | 9.23 | 8.85 | 7.99 | 7.99 | 8.92 | 9.23 | 10.80 | 10.30 | 9.47 | 11.70 | 8.14 | 9.08 | 9.52 |
| A218 | 10.20 | 9.14 | 9.03 | 11.30 | 9.65 | 10.70 | 10.50 | 10.70 | 11.00 | 12.50 | 12.50 | 10.70 | 10.30 | 12.70 |
| A219 | 10.20 | 9.39 | 9.66 | 10.80 | 9.85 | 10.90 | 11.30 | 11.50 | 11.80 | 13.90 | 13.10 | 10.20 | 10.80 | 14.30 |
| M221 | 8.32 | 7.06 | 6.00 | 7.55 | 5.65 | 9.94 | 8.42 | 10.30 | 8.80 | 9.61 | 11.80 | 6.80 | 9.88 | 9.84 |
| L222 | 9.58 | 9.89 | 9.35 | 9.40 | 9.27 | 10.00 | 10.20 | 10.70 | 10.80 | 10.30 | 10.90 | 9.33 | 9.90 | 10.50 |
| L223 | 10.40 | 9.88 | 9.78 | 10.70 | 10.50 | 11.30 | 11.00 | 11.50 | 12.90 | 13.00 | 13.30 | 10.10 | 10.90 | 13.20 |
| R224 | 8.10 | 8.61 | 8.10 | 7.94 | 2.34 | 7.70 | 7.57 | 8.59 | 10.60 | 10.40 | 10.90 | 8.99 | 8.19 | 10.30 |
| V225 | 9.13 | 9.04 | 9.91 | 8.86 | 8.92 | 11.10 | 10.10 | 9.56 | 10.70 | 12.10 | 11.30 | 9.69 | 9.63 | 12.10 |
| T227 | 6.36 | 5.50 | 5.59 | 7.49 | 6.03 | 9.35 | 8.86 | 7.62 | 7.32 | 8.20 | 9.06 | 7.06 | 7.74 | 8.43 |
| V228 | 4.41 | 4.03 | 5.10 | 4.15 | 4.63 | 5.91 | 4.19 | 4.36 | 6.78 | 6.53 | 7.35 | 4.45 | 5.22 | 6.44 |
| L230 | 0.15 | 1.66 | 0.21 | 0.85 | 0.56 | 1.48 | 1.59 | 2.60 | 3.21 | 4.06 | 4.38 | 1.81 | 2.66 | 4.39 |
| A232 | 0.91 | 0.82 | 0.50 | 1.24 | 1.04 | 1.98 | 2.04 | 2.67 | 3.21 | 5.87 | 5.78 | 0.57 | 2.64 | 4.22 |

**Supplementary File 1g**: *R_1ρ_* relaxation rates measured using HSn pulses (n=1,2,4,6,8) from HARD experiment for apo TRBP2-dsRBD2 at 600 MHz NMR spectrometer.

| **Residue**  **Number** | ***R_1ρ_* (s^-1^)** | | | | | | | | | |
| --- | --- | --- | --- | --- | --- | --- | --- | --- | --- | --- |
|  | **HS1** | | **HS2** | | **HS4** | | **HS6** | | **HS8** | |
|  | **Value** | **Error** | **Value** | **Error** | **Value** | **Error** | **Value** | **Error** | **Value** | **Error** |
| S151 | 3.76 | 0.39 | 3.67 | 0.29 | 4.18 | 0.25 | 4.01 | 0.12 | 4.86 | 0.54 |
| Q154 | 4.78 | 0.30 | 4.40 | 0.30 | 4.93 | 0.36 | 5.19 | 0.13 | 6.07 | 0.42 |
| Q155 | 5.14 | 0.23 | 5.02 | 0.32 | 5.13 | 0.43 | 5.51 | 0.10 | 6.06 | 0.28 |
| S156 | 4.37 | 0.53 | 4.26 | 0.33 | 5.08 | 0.32 | 4.78 | 0.18 | 5.87 | 0.44 |
| E157 | 4.68 | 0.38 | 4.34 | 0.25 | 4.94 | 0.31 | 5.02 | 0.11 | 6.02 | 0.48 |
| N159 | 4.12 | 0.56 | 4.06 | 0.45 | 4.42 | 0.19 | 5.01 | 0.25 | 5.92 | 0.50 |
| G162 | 4.75 | 0.27 | 5.34 | 0.29 | 6.05 | 0.14 | 6.31 | 0.13 | 7.00 | 0.39 |
| A163 | 5.54 | 0.44 | 5.55 | 0.42 | 6.81 | 0.29 | 6.80 | 0.16 | 8.30 | 0.64 |
| Q165 | 4.84 | 0.39 | 5.16 | 0.32 | 6.06 | 0.16 | 6.32 | 0.15 | 7.44 | 0.43 |
| E166 | 4.86 | 0.34 | 5.54 | 0.24 | 6.35 | 0.23 | 6.44 | 0.10 | 7.60 | 0.40 |
| L167 | 5.35 | 0.23 | 5.87 | 0.29 | 6.88 | 0.35 | 6.99 | 0.19 | 7.95 | 0.43 |
| V168 | 5.82 | 0.46 | 6.73 | 0.35 | 8.11 | 0.27 | 8.62 | 0.25 | 9.90 | 0.49 |
| V169 | 4.79 | 0.28 | 5.05 | 0.34 | 6.09 | 0.27 | 6.57 | 0.21 | 7.40 | 0.29 |
| Q170 | 5.78 | 0.42 | 6.29 | 0.23 | 6.94 | 0.33 | 7.34 | 0.15 | 8.34 | 0.39 |
| G172 | 4.34 | 0.32 | 4.35 | 0.31 | 5.27 | 0.21 | 5.57 | 0.09 | 6.42 | 0.34 |
| W173 | 4.70 | 0.35 | 4.97 | 0.17 | 5.91 | 0.23 | 5.88 | 0.13 | 6.71 | 0.43 |
| E177 | 3.96 | 0.25 | 4.01 | 0.25 | 4.89 | 0.22 | 4.92 | 0.10 | 5.70 | 0.36 |
| Y178 | 4.05 | 0.29 | 4.33 | 0.23 | 4.88 | 0.16 | 5.27 | 0.11 | 5.95 | 0.37 |
| T179 | 4.31 | 0.32 | 5.02 | 0.25 | 5.53 | 0.14 | 5.86 | 0.13 | 6.65 | 0.35 |
| V180 | 4.67 | 0.33 | 4.57 | 0.19 | 5.94 | 0.29 | 6.00 | 0.15 | 6.69 | 0.47 |
| T181 | 3.86 | 0.26 | 4.35 | 0.22 | 5.09 | 0.17 | 5.18 | 0.11 | 5.91 | 0.29 |
| Q182 | 5.05 | 0.34 | 5.59 | 0.24 | 6.12 | 0.27 | 6.42 | 0.10 | 7.41 | 0.36 |
| E183 | 4.67 | 0.32 | 4.72 | 0.36 | 5.66 | 0.26 | 5.57 | 0.11 | 6.49 | 0.41 |
| G185 | 5.01 | 0.25 | 5.02 | 0.30 | 5.75 | 0.32 | 5.56 | 0.27 | 6.71 | 0.35 |
| R189 | 4.72 | 0.45 | 4.91 | 0.27 | 6.38 | 0.13 | 6.23 | 0.18 | 7.60 | 0.50 |
| E191 | 4.06 | 0.32 | 4.03 | 0.26 | 4.88 | 0.25 | 5.07 | 0.10 | 5.95 | 0.40 |
| T193 | 4.49 | 0.31 | 5.08 | 0.23 | 5.85 | 0.24 | 5.99 | 0.11 | 6.91 | 0.33 |
| M194 | 4.88 | 0.18 | 4.94 | 0.36 | 6.04 | 0.26 | 6.12 | 0.26 | 6.44 | 0.58 |
| T195 | 4.96 | 0.29 | 5.35 | 0.19 | 6.21 | 0.21 | 6.31 | 0.07 | 7.24 | 0.34 |
| C196 | 4.51 | 0.36 | 5.17 | 0.33 | 5.68 | 0.14 | 5.93 | 0.18 | 6.79 | 0.37 |
| R197 | 5.29 | 0.32 | 5.36 | 0.20 | 6.01 | 0.36 | 6.43 | 0.22 | 6.90 | 0.34 |
| V198 | 3.99 | 0.48 | 4.03 | 0.10 | 4.92 | 0.22 | 5.12 | 0.24 | 5.77 | 0.33 |
| E199 | 4.53 | 0.37 | 4.68 | 0.28 | 5.62 | 0.26 | 5.62 | 0.12 | 6.74 | 0.41 |
| R200 | 4.33 | 0.29 | 5.36 | 0.36 | 6.01 | 0.35 | 6.09 | 0.23 | 7.10 | 0.29 |
| F201 | 4.96 | 0.35 | 5.18 | 0.25 | 6.02 | 0.28 | 6.26 | 0.13 | 6.96 | 0.35 |
| I202 | 4.49 | 0.24 | 4.98 | 0.23 | 5.67 | 0.19 | 5.89 | 0.20 | 6.54 | 0.32 |
| E203 | 5.04 | 0.29 | 5.33 | 0.24 | 5.89 | 0.22 | 6.00 | 0.12 | 6.95 | 0.34 |
| I204 | 5.08 | 0.27 | 5.51 | 0.17 | 6.11 | 0.37 | 6.07 | 0.11 | 7.13 | 0.24 |
| G205 | 4.37 | 0.27 | 4.91 | 0.19 | 5.68 | 0.25 | 6.04 | 0.14 | 6.68 | 0.32 |
| S206 | 4.29 | 0.36 | 4.36 | 0.27 | 5.13 | 0.14 | 5.37 | 0.12 | 6.08 | 0.38 |
| G207 | 4.48 | 0.34 | 4.76 | 0.32 | 5.42 | 0.20 | 5.87 | 0.11 | 6.73 | 0.45 |
| T208 | 5.53 | 0.47 | 5.54 | 0.46 | 6.29 | 0.27 | 6.33 | 0.33 | 7.28 | 0.49 |
| S209 | 3.64 | 0.36 | 3.38 | 0.50 | 4.04 | 0.59 | 4.15 | 0.46 | 5.07 | 0.44 |
| L212 | 4.76 | 0.34 | 4.97 | 0.35 | 6.13 | 0.23 | 6.20 | 0.26 | 7.52 | 0.35 |
| A213 | 4.74 | 0.46 | 4.83 | 0.26 | 5.81 | 0.19 | 6.16 | 0.14 | 7.21 | 0.54 |
| R215 | 4.81 | 0.46 | 5.38 | 0.35 | 6.39 | 0.21 | 6.76 | 0.29 | 7.82 | 0.58 |
| N216 | 4.95 | 0.40 | 5.55 | 0.36 | 6.48 | 0.24 | 6.64 | 0.19 | 7.76 | 0.46 |
| A217 | 4.34 | 0.44 | 4.93 | 0.27 | 6.06 | 0.22 | 5.91 | 0.22 | 7.05 | 0.40 |
| A218 | 4.93 | 0.36 | 5.26 | 0.35 | 6.16 | 0.21 | 6.64 | 0.29 | 7.68 | 0.54 |
| A219 | 4.67 | 0.36 | 5.03 | 0.41 | 6.01 | 0.20 | 6.26 | 0.19 | 7.42 | 0.44 |
| L223 | 4.88 | 0.35 | 5.64 | 0.18 | 6.50 | 0.31 | 6.74 | 0.20 | 7.79 | 0.34 |
| R224 | 4.56 | 0.22 | 4.66 | 0.34 | 5.56 | 0.21 | 5.91 | 0.21 | 6.93 | 0.58 |
| V225 | 5.57 | 0.24 | 5.62 | 0.32 | 6.48 | 0.36 | 6.74 | 0.21 | 7.56 | 0.63 |
| T227 | 4.44 | 0.25 | 4.70 | 0.19 | 5.44 | 0.34 | 5.72 | 0.14 | 6.65 | 0.44 |
| V228 | 5.38 | 0.24 | 5.29 | 0.24 | 5.89 | 0.31 | 5.83 | 0.08 | 6.96 | 0.46 |
| L230 | 3.33 | 0.43 | 2.93 | 0.33 | 3.54 | 0.19 | 3.51 | 0.18 | 4.33 | 0.57 |
| A232 | 4.65 | 0.40 | 4.27 | 0.31 | 4.83 | 0.25 | 4.83 | 0.17 | 5.84 | 0.57 |

**Supplementary File 1h**: *R_2ρ_* relaxation rates measured using HSn pulses (n=1, 2, 4, 6, 8) from HARD experiment for apo TRBP2-dsRBD2 at 600 MHz NMR spectrometer.

| **Residue Number** | ***R_2ρ_* (s^-1^)** | | | | | | | | | |
| --- | --- | --- | --- | --- | --- | --- | --- | --- | --- | --- |
|  | **HS1** | | **HS2** | | **HS4** | | **HS6** | | **HS8** | |
|  | **Value** | **Error** | **Value** | **Error** | **Value** | **Error** | **Value** | **Error** | **Value** | **Error** |
| S151 | 7.22 | 0.25 | 7.20 | 0.35 | 6.78 | 0.07 | 7.27 | 0.19 | 7.30 | 0.64 |
| Q154 | 6.78 | 0.24 | 6.98 | 0.15 | 6.55 | 0.36 | 6.54 | 0.35 | 7.20 | 0.22 |
| Q155 | 5.75 | 0.74 | 5.06 | 0.11 | 4.86 | 0.18 | 6.35 | 1.02 | 6.09 | 0.41 |
| S156 | 9.58 | 0.68 | 9.00 | 0.63 | 8.43 | 0.51 | 7.46 | 0.45 | 8.63 | 0.49 |
| E157 | 7.68 | 0.72 | 7.75 | 0.13 | 7.05 | 0.45 | 7.44 | 0.81 | 7.78 | 0.40 |
| N159 | 12.73 | 0.23 | 14.44 | 0.79 | 12.52 | 1.01 | 10.49 | 0.77 | 14.41 | 0.99 |
| G162 | 12.32 | 0.37 | 12.14 | 0.46 | 12.59 | 0.03 | 12.88 | 0.09 | 12.30 | 0.35 |
| A163 | 13.65 | 0.91 | 13.41 | 0.62 | 11.97 | 0.31 | 12.47 | 1.28 | 14.41 | 0.35 |
| L164 | 14.55 | 0.24 | 13.28 | 0.30 | 13.33 | 0.16 | 13.83 | 0.03 | 12.48 | 0.32 |
| Q165 | 14.05 | 0.55 | 13.32 | 0.41 | 12.93 | 0.65 | 13.00 | 0.78 | 13.00 | 0.18 |
| E166 | 12.61 | 0.35 | 12.51 | 0.03 | 11.74 | 0.28 | 12.00 | 0.51 | 12.45 | 0.12 |
| L167 | 13.39 | 0.76 | 12.91 | 0.35 | 12.96 | 0.25 | 13.32 | 0.41 | 12.99 | 0.86 |
| V168 | 20.39 | 1.92 | 19.34 | 0.25 | 16.87 | 0.79 | 18.63 | 2.04 | 19.05 | 0.87 |
| V169 | 14.19 | 0.95 | 13.55 | 0.54 | 12.16 | 0.88 | 12.45 | 0.95 | 14.13 | 0.70 |
| Q170 | 13.85 | 0.07 | 13.71 | 0.50 | 13.03 | 0.30 | 13.10 | 0.39 | 13.28 | 0.49 |
| G172 | 12.51 | 0.49 | 11.90 | 0.31 | 12.13 | 0.76 | 11.81 | 0.96 | 11.88 | 0.14 |
| W173 | 11.85 | 0.39 | 11.63 | 0.59 | 11.42 | 0.25 | 10.98 | 0.69 | 11.25 | 0.26 |
| R174 | 11.73 | 0.24 | 10.27 | 0.78 | 10.40 | 0.68 | 10.23 | 0.49 | 9.29 | 0.15 |
| E177 | 10.83 | 0.46 | 10.81 | 0.18 | 10.60 | 0.31 | 10.36 | 0.32 | 10.61 | 0.33 |
| Y178 | 11.61 | 0.04 | 10.91 | 0.37 | 10.97 | 0.47 | 10.72 | 0.55 | 10.75 | 0.39 |
| T179 | 11.63 | 0.34 | 11.39 | 0.59 | 11.19 | 0.21 | 11.14 | 0.27 | 11.19 | 0.37 |
| V180 | 15.96 | 0.78 | 14.19 | 1.10 | 13.22 | 0.40 | 13.27 | 0.85 | 13.50 | 0.63 |
| T181 | 11.86 | 0.29 | 11.37 | 0.22 | 10.92 | 0.12 | 10.72 | 0.37 | 11.02 | 0.37 |
| Q182 | 12.69 | 0.18 | 12.46 | 0.52 | 12.48 | 0.22 | 12.44 | 0.27 | 12.36 | 0.44 |
| E183 | 11.39 | 0.60 | 10.75 | 0.20 | 10.48 | 0.48 | 10.83 | 0.68 | 10.99 | 0.46 |
| G185 | 9.43 | 0.58 | 9.59 | 0.34 | 8.82 | 0.46 | 9.58 | 0.32 | 10.15 | 0.25 |
| R189 | 15.64 | 1.98 | 14.98 | 0.94 | 14.67 | 0.66 | 13.13 | 1.48 | 13.28 | 1.04 |
| E191 | 10.71 | 0.42 | 9.96 | 0.37 | 9.94 | 0.21 | 10.04 | 0.31 | 9.51 | 0.12 |
| F192 | 13.38 | 0.07 | 12.92 | 0.36 | 13.11 | 0.10 | 12.54 | 0.36 | 12.90 | 0.12 |
| T193 | 12.72 | 0.50 | 12.80 | 0.72 | 12.45 | 0.48 | 12.58 | 0.32 | 12.45 | 0.66 |
| M194 | 8.43 | 0.49 | 9.32 | 1.43 | 8.84 | 0.51 | 9.21 | 0.28 | 10.98 | 1.37 |
| T195 | 13.09 | 0.38 | 13.14 | 0.79 | 13.18 | 0.39 | 13.38 | 0.18 | 12.72 | 0.47 |
| C196 | 12.58 | 0.35 | 12.68 | 0.32 | 11.60 | 0.80 | 10.74 | 0.74 | 12.52 | 0.86 |
| R197 | 10.37 | 0.70 | 11.05 | 1.01 | 10.43 | 0.25 | 10.60 | 0.96 | 11.38 | 0.36 |
| V198 | 13.26 | 1.36 | 11.51 | 1.40 | 12.37 | 1.02 | 10.36 | 1.86 | 10.64 | 0.94 |
| E199 | 11.88 | 0.19 | 11.36 | 0.19 | 11.15 | 0.13 | 11.10 | 0.51 | 11.18 | 0.29 |
| R200 | 12.68 | 2.54 | 10.82 | 1.71 | 10.99 | 1.47 | 11.10 | 1.08 | 11.60 | 2.00 |
| F201 | 11.48 | 0.45 | 11.38 | 0.11 | 10.67 | 0.38 | 10.77 | 0.58 | 11.34 | 0.09 |
| I202 | 11.36 | 0.79 | 11.19 | 0.64 | 10.99 | 0.51 | 10.34 | 0.47 | 10.68 | 0.16 |
| E203 | 11.67 | 0.56 | 12.18 | 0.20 | 10.82 | 0.26 | 10.92 | 0.62 | 11.83 | 0.32 |
| I204 | 10.53 | 0.64 | 11.15 | 0.29 | 10.62 | 0.32 | 10.66 | 0.48 | 10.75 | 0.29 |
| G205 | 11.98 | 0.40 | 11.92 | 0.21 | 11.30 | 0.23 | 11.16 | 0.48 | 11.37 | 0.07 |
| S206 | 11.12 | 0.13 | 10.59 | 0.33 | 10.53 | 0.05 | 11.01 | 0.14 | 10.66 | 0.15 |
| G207 | 13.23 | 0.39 | 12.35 | 0.42 | 12.69 | 0.53 | 12.58 | 0.86 | 12.33 | 0.29 |
| T208 | 12.86 | 0.74 | 12.63 | 0.55 | 11.74 | 0.46 | 12.86 | 0.66 | 11.34 | 0.23 |
| S209 | 9.60 | 1.46 | 9.31 | 0.23 | 11.16 | 0.32 | 10.41 | 0.63 | 11.80 | 1.09 |
| L212 | 14.93 | 1.52 | 14.22 | 0.75 | 13.55 | 0.97 | 12.83 | 0.96 | 13.68 | 0.85 |
| A213 | 12.78 | 0.53 | 11.71 | 0.50 | 12.23 | 0.90 | 12.87 | 0.12 | 12.54 | 0.72 |
| R215 | 14.78 | 1.64 | 13.74 | 1.27 | 14.28 | 0.93 | 13.86 | 0.40 | 14.89 | 1.57 |
| N216 | 14.09 | 0.92 | 14.09 | 0.39 | 12.96 | 0.44 | 12.77 | 0.70 | 13.37 | 0.41 |
| A217 | 13.10 | 0.42 | 12.05 | 0.69 | 12.37 | 0.47 | 12.54 | 0.89 | 12.05 | 0.10 |
| A218 | 14.84 | 0.80 | 13.03 | 0.29 | 13.80 | 0.43 | 14.71 | 0.56 | 13.03 | 0.47 |
| A219 | 14.93 | 0.28 | 13.18 | 0.34 | 13.53 | 0.40 | 13.84 | 0.49 | 12.58 | 0.37 |
| L222 | 13.60 | 0.24 | 13.10 | 0.38 | 12.83 | 0.05 | 12.41 | 0.37 | 12.52 | 0.13 |
| L223 | 13.32 | 0.20 | 13.13 | 1.16 | 12.79 | 0.21 | 13.05 | 0.35 | 13.32 | 0.69 |
| R224 | 15.29 | 2.02 | 14.00 | 1.41 | 14.32 | 0.30 | 13.10 | 0.98 | 12.28 | 1.27 |
| V225 | 12.72 | 1.02 | 12.95 | 0.62 | 12.63 | 0.80 | 12.77 | 0.43 | 12.13 | 1.31 |
| T227 | 10.96 | 1.32 | 11.29 | 0.74 | 10.76 | 1.18 | 10.05 | 1.02 | 10.45 | 0.41 |
| V228 | 9.46 | 0.64 | 9.48 | 0.49 | 8.37 | 0.61 | 8.64 | 0.76 | 9.16 | 0.61 |
| L230 | 4.07 | 0.15 | 3.97 | 0.17 | 4.03 | 0.18 | 4.51 | 0.46 | 4.65 | 0.14 |
| A232 | 6.18 | 0.31 | 6.18 | 0.21 | 5.37 | 0.30 | 5.70 | 0.80 | 6.94 | 0.23 |

**Supplementary File 1i:** Dynamics parameters extracted from HARD experimental data from geoHARD method for apo TRBP2-dsRBD2.

| **Residue Number** | ***k_ex_* (Hz)** | | ***p_B_* (%)** |
| --- | --- | --- | --- |
|  | **Value** | **Error** |  |
| N159 | 193.02 | 74.20 | 2.18 |
| G162 | 108.91 | 9.01 | 1.39 |
| A163 | 143.20 | 40.06 | 1.10 |
| Q165 | 145.51 | 32.02 | 1.60 |
| E166 | 113.27 | 11.37 | 0.94 |
| L167 | 153.11 | 78.02 | 0.63 |
| V169 | 181.02 | 63.29 | 1.41 |
| G172 | 149.65 | 32.10 | 1.64 |
| W173 | 111.43 | 13.12 | 0.99 |
| E177 | 144.32 | 24.23 | 1.43 |
| Y178 | 149.17 | 45.08 | 1.34 |
| T179 | 116.90 | 6.58 | 1.05 |
| V180 | 30162.81 | 2556.08 | 4.98 |
| T181 | 225.86 | 113.77 | 1.16 |
| Q182 | 104.47 | 6.91 | 1.12 |
| E183 | 151.01 | 54.24 | 0.92 |
| R189 | 31748.72 | 955.20 | 9.37 |
| E191 | 185.94 | 58.22 | 0.89 |
| T193 | 113.50 | 14.73 | 1.51 |
| T195 | 110.02 | 13.94 | 1.60 |
| C196 | 164.04 | 75.77 | 1.18 |
| V198 | 30554.87 | 1687.74 | 3.05 |
| E199 | 115.53 | 14.31 | 1.31 |
| R200 | 53667.71 | 2331.35 | 15.82 |
| F201 | 148.85 | 63.15 | 0.56 |
| I202 | 153.01 | 64.86 | 0.74 |
| E203 | 129.64 | 34.33 | 0.84 |
| 205 | 153.79 | 40.99 | 0.96 |
| S206 | 137.90 | 32.54 | 1.26 |
| G207 | 204.01 | 64.95 | 1.34 |
| T208 | 173.36 | 69.90 | 0.81 |
| S209 | 110.46 | 12.18 | 2.16 |
| L212 | 36081.01 | 4503.77 | 10.56 |
| A213 | 121.17 | 17.10 | 1.53 |
| N216 | 195.81 | 71.97 | 1.07 |
| A217 | 160.24 | 74.26 | 1.40 |
| A218 | 162.39 | 53.99 | 1.45 |
| A219 | 138.44 | 43.89 | 1.74 |
| L223 | 107.89 | 13.48 | 0.95 |
| R224 | 31519.02 | 1155.44 | 15.57 |
| T227 | 52411.81 | 1883.58 | 7.57 |

**Supplementary File 1j**: ITC binding study of TRBP2-dsRBD2 and D12 RNA carried out in triplicate.

| Experiment No. | [Syr]  (mM)  TRBP2_dsRBD2 | [Cell]  (μM)  D12 RNA | n (stoichiometry) | K_d_ (μM) | ΔH (kcal/mol) |
| --- | --- | --- | --- | --- | --- |
| 1) | 1.4 | 10 | 3.03 ± 0.06 | 1.13 ± 0.19 | -9.2 ± 0.23 |
| 2) | 0.8 | 10 | 2.84 ± 0.09 | 0.996 ± 0.31 | -9.16 ± 0.39 |
| 3) | 0.8 | 10 | 2.59 ± 0.11 | 1.41 ± 0.46 | -12 ± 0.67 |
| **Average** | - | - | **2.82 ± 0.09** | **1.18 ± 0.32** | **-10.12 ± 0.43** |

**Supplementary File 1k**: Nuclear spin relaxation data for RNA-bound TRBP2-dsRBD2 recorded at 600 MHz and 800 MHz NMR spectrometer. Data for some residues is missing in this table due to line-broadening issues in the corresponding experiments.

| **Residue Number** | **600 MHz** | | | | | | **800 MHz** | | | | | |
| --- | --- | --- | --- | --- | --- | --- | --- | --- | --- | --- | --- | --- |
|  | ***R_1_* (s^-1^)** | | ***R_2_* (s^-1^)** | | **NOE** | | ***R_1_* (s^-1^)** | | ***R_2_* (s^-1^)** | | **NOE** | |
|  | **Value** | **Error** | **Value** | **Error** | **Value** | **Error** | **Value** | **Error** | **Value** | **Error** | **Value** | **Error** |
| S151 | 1.48 | 0.10 | 7.55 | 0.29 | -0.26 | -0.02 | 1.32 | 0.25 | 7.24 | 0.30 | 0.10 | 0.03 |
| Q154 | 1.57 | 0.02 | 6.12 | 0.06 | - | - | 1.40 | 0.13 | 5.76 | 0.25 | 0.38 | 0.01 |
| Q155 | 1.39 | 0.03 | 4.68 | 0.10 | - | - | 1.25 | 0.14 | 5.54 | 0.09 | - | - |
| S156 | 1.50 | 0.13 | 10.32 | 0.26 | 0.04 | 0.02 | 1.39 | 0.27 | 11.19 | 0.26 | 0.33 | 0.03 |
| E157 | 1.74 | 0.11 | 11.13 | 0.28 | 0.26 | 0.02 | 1.45 | 0.22 | 11.59 | 0.41 | 0.39 | 0.03 |
| N159 | 1.39 | 0.15 | 17.27 | 1.13 | 0.38 | 0.07 | 1.54 | 0.27 | 21.17 | 1.04 | 0.55 | 0.14 |
| G162 | 1.32 | 0.07 | 23.58 | 0.97 | 0.85 | 0.07 | 1.19 | 0.10 | 24.71 | 2.09 | 0.81 | 0.15 |
| A163 | 1.28 | 0.10 | 22.33 | 2.50 | 0.86 | 0.09 | 0.88 | 0.12 | 25.06 | 2.61 | 1.01 | 0.16 |
| Q165 | 1.29 | 0.06 | 24.36 | 0.97 | 0.76 | 0.05 | 1.02 | 0.10 | 24.87 | 0.54 | 0.85 | 0.07 |
| E166 | 1.35 | 0.04 | 23.64 | 0.74 | 0.74 | 0.03 | 1.04 | 0.09 | 24.39 | 0.35 | 0.77 | 0.06 |
| L167 | 1.34 | 0.06 | 22.40 | 0.39 | 0.76 | 0.07 | 0.86 | 0.07 | 26.05 | 0.62 | 0.73 | 0.13 |
| V168 | 1.27 | 0.07 | 24.52 | 3.18 | 0.71 | 0.11 | 0.96 | 0.17 | 33.08 | 2.23 | 0.63 | 0.22 |
| V169 | 1.27 | 0.10 | 14.23 | 1.55 | 0.77 | 0.17 | 1.16 | 0.10 | 12.32 | 1.64 | 0.69 | 0.22 |
| Q170 | 1.29 | 0.05 | 22.32 | 0.55 | 0.74 | 0.03 | 1.01 | 0.07 | 23.28 | 0.82 | 0.76 | 0.06 |
| G172 | 1.38 | 0.04 | 23.28 | 0.60 | 0.77 | 0.04 | 1.00 | 0.09 | 27.34 | 1.36 | 0.68 | 0.07 |
| W173 | 1.41 | 0.03 | 19.33 | 0.84 | 0.74 | 0.05 | 1.01 | 0.07 | 21.83 | 0.84 | 0.82 | 0.07 |
| E177 | 1.30 | 0.05 | 7.35 | 0.79 | 0.31 | 0.02 | 1.11 | 0.15 | 8.38 | 0.93 | 0.66 | 0.05 |
| Y178 | 1.43 | 0.04 | 21.96 | 1.13 | 0.74 | 0.06 | 0.89 | 0.08 | 22.49 | 2.11 | 0.93 | 0.11 |
| T179 | 1.38 | 0.06 | 20.72 | 0.42 | 0.82 | 0.05 | 0.90 | 0.17 | 23.41 | 0.64 | 0.68 | 0.09 |
| V180 | 1.77 | 0.14 | 20.46 | 1.68 | 0.67 | 0.10 | 1.16 | 0.30 | 25.52 | 2.19 | 1.22 | 0.30 |
| Q182 | 1.47 | 0.05 | 19.91 | 0.63 | 0.77 | 0.04 | 1.26 | 0.12 | 25.21 | 1.67 | 0.74 | 0.09 |
| E183 | 1.45 | 0.04 | 17.79 | 0.36 | 0.67 | 0.03 | 1.24 | 0.14 | 20.92 | 0.40 | 0.71 | 0.05 |
| G185 | 1.64 | 0.04 | 15.33 | 0.53 | 0.57 | 0.05 | 1.20 | 0.19 | 20.94 | 0.54 | 0.68 | 0.07 |
| R189 | 1.74 | 0.10 | - | - | 0.34 | 0.08 | 1.41 | 0.09 | 26.32 | 1.69 | 0.90 | 0.25 |
| E191 | 1.33 | 0.07 | 17.75 | 0.33 | 0.56 | 0.04 | 0.94 | 0.14 | 19.38 | 0.68 | 0.52 | 0.06 |
| T193 | 1.20 | 0.08 | 21.32 | 1.07 | 0.69 | 0.07 | 1.05 | 0.15 | 22.19 | 0.95 | 0.67 | 0.10 |
| M194 | 1.49 | 0.13 | 22.38 | 2.67 | 0.89 | 0.18 | 1.28 | 0.06 | 21.07 | 4.10 | 0.77 | 0.26 |
| T195 | 1.33 | 0.05 | 23.04 | 0.64 | 0.84 | 0.07 | 1.06 | 0.15 | 27.43 | 1.58 | 0.87 | 0.14 |
| C196 | 1.30 | 0.15 | 20.41 | 1.26 | 0.59 | 0.10 | 1.20 | 0.14 | 25.47 | 1.26 | 0.55 | 0.11 |
| R197 | 1.20 | 0.14 | 22.37 | 2.66 | 1.05 | 0.19 | 1.38 | 0.13 | 26.97 | 1.80 | 0.76 | 0.21 |
| V198 | 1.15 | 0.10 | 19.37 | 1.51 | 0.77 | 0.14 | 1.03 | 0.20 | 24.74 | 3.10 | 1.08 | 0.29 |
| E199 | 1.39 | 0.05 | 18.73 | 0.55 | 0.70 | 0.04 | 1.02 | 0.07 | 20.50 | 0.76 | 0.78 | 0.08 |
| R200 | 1.30 | 0.11 | 17.60 | 0.98 | 0.80 | 0.13 | 1.16 | 0.10 | 20.62 | 2.50 | 0.59 | 0.18 |
| F201 | 1.41 | 0.06 | 20.46 | 0.67 | 0.74 | 0.04 | 1.00 | 0.08 | 22.79 | 0.92 | 0.76 | 0.06 |
| I202 | 1.33 | 0.05 | 19.62 | 0.78 | 0.69 | 0.05 | 1.00 | 0.09 | 20.53 | 0.59 | 0.84 | 0.10 |
| E203 | 1.46 | 0.02 | 19.29 | 0.65 | 0.77 | 0.04 | 0.99 | 0.10 | 24.02 | 0.46 | 0.77 | 0.08 |
| I204 | 1.22 | 0.05 | 20.52 | 1.00 | 0.76 | 0.08 | 0.86 | 0.09 | 22.86 | 0.81 | 0.87 | 0.15 |
| G205 | 1.52 | 0.05 | 19.70 | 0.73 | 0.85 | 0.06 | 1.11 | 0.16 | 19.37 | 0.87 | 1.02 | 0.13 |
| S206 | 1.36 | 0.04 | 20.38 | 0.54 | 0.74 | 0.03 | 0.95 | 0.13 | 20.90 | 0.65 | 0.80 | 0.06 |
| G207 | 1.35 | 0.04 | 22.78 | 0.80 | 0.77 | 0.04 | 1.00 | 0.12 | 22.56 | 0.79 | 0.97 | 0.10 |
| T208 | 1.61 | 0.10 | 19.56 | 0.63 | 0.69 | 0.05 | 1.31 | 0.24 | 22.45 | 1.75 | 0.94 | 0.12 |
| S209 | 1.32 | 0.07 | 10.29 | 2.68 | 0.11 | 0.04 | 1.06 | 0.15 | 7.85 | 5.43 | 0.49 | 0.17 |
| L212 | 1.07 | 0.14 | 22.60 | 2.24 | 0.97 | 0.11 | 0.76 | 0.22 | 23.43 | 1.98 | 0.86 | 0.17 |
| A213 | 1.43 | 0.05 | 17.74 | 1.45 | 0.82 | 0.09 | 0.98 | 0.11 | 20.26 | 1.86 | 0.95 | 0.17 |
| R215 | 1.37 | 0.12 | 20.01 | 2.07 | 1.03 | 0.15 | 0.92 | 0.05 | 25.59 | 1.33 | 0.77 | 0.15 |
| N216 | 1.26 | 0.05 | 24.39 | 0.72 | 0.75 | 0.04 | 0.87 | 0.10 | 24.44 | 0.92 | 0.77 | 0.07 |
| A217 | 1.39 | 0.03 | 18.58 | 1.56 | 0.64 | 0.08 | 1.06 | 0.10 | 23.62 | 1.23 | 1.08 | 0.21 |
| A219 | 1.37 | 0.05 | 24.46 | 0.93 | 0.83 | 0.06 | 1.01 | 0.01 | 27.16 | 0.21 | 0.72 | 0.13 |
| L223 | 1.18 | 0.03 | 24.26 | 0.56 | 0.74 | 0.07 | 0.92 | 0.06 | 27.62 | 0.81 | 0.85 | 0.10 |
| R224 | 1.47 | 0.13 | 19.59 | 1.09 | 0.84 | 0.14 | 0.95 | 0.16 | 25.31 | 2.80 | 0.74 | 0.18 |
| V225 | 1.43 | 0.09 | 18.70 | 1.00 | 0.75 | 0.14 | 0.95 | 0.14 | 23.69 | 2.26 | 0.81 | 0.22 |
| T227 | 1.70 | 0.10 | 15.33 | 0.31 | 0.53 | 0.03 | 1.36 | 0.18 | 16.08 | 0.24 | 0.64 | 0.05 |
| V228 | 1.52 | 0.05 | 11.29 | 0.20 | 0.48 | 0.04 | 1.31 | 0.12 | 13.59 | 0.38 | 0.63 | 0.06 |
| L230 | 0.97 | 0.01 | 4.70 | 0.09 | -0.93 | -0.01 | 1.02 | 0.04 | 2.33 | 0.60 | -0.63 | -0.01 |
| A232 | 1.35 | 0.04 | 4.85 | 0.19 | -0.25 | -0.02 | 1.29 | 0.14 | 5.94 | 0.37 | 0.29 | 0.03 |

**Supplementary File 1l**: Order parameter (S^2^) extracted from Model-free analysis of the Nuclear Spin Relaxation data for RNA-bound TRBP2-dsRBD2 recorded at 600 MHz and 800 MHz NMR spectrometer.

| **Residue Number** | **S^2^** | |
| --- | --- | --- |
|  | **Value** | **Error** |
| N159 | 0.22 | 0.01 |
| G162 | 0.98 | 0.02 |
| A163 | 0.27 | 0.08 |
| Q165 | 0.79 | 0.01 |
| V168 | 0.92 | 0.04 |
| V169 | 0.38 | 0.04 |
| Q170 | 0.96 | 0.02 |
| G172 | 0.93 | 0.02 |
| W173 | 0.82 | 0.03 |
| Y178 | 0.94 | 0.02 |
| T179 | 0.86 | 0.01 |
| Q182 | 0.57 | 0.02 |
| E183 | 0.31 | 0.01 |
| E191 | 0.29 | 0.06 |
| T193 | 0.84 | 0.03 |
| M194 | 0.88 | 0.04 |
| T195 | 0.81 | 0.03 |
| V198 | 0.61 | 0.05 |
| R200 | 0.49 | 0.03 |
| F201 | 0.89 | 0.02 |
| I202 | 0.72 | 0.02 |
| E203 | 0.94 | 0.01 |
| I204 | 0.83 | 0.03 |
| G205 | 0.65 | 0.03 |
| S206 | 0.89 | 0.02 |
| G207 | 0.81 | 0.03 |
| T208 | 0.92 | 0.03 |
| L212 | 0.93 | 0.04 |
| A213 | 0.94 | 0.03 |
| N216 | 0.85 | 0.03 |
| A219 | 0.91 | 0.01 |
| L223 | 0.90 | 0.02 |
| R224 | 0.78 | 0.03 |
| V225 | 0.64 | 0.04 |

**Supplementary File 1m***: R_ex_* of residues extracted from Model-free analysis of Nuclear Spin Relaxation data for RNA-bound TRBP2-dsRBD2 recorded at 600 MHz and 800 MHz NMR spectrometer.

| **Residue Number** | ***R_ex_* (s^-1^)** | |
| --- | --- | --- |
|  | **Value** | **Error** |
| V168 | 4.70 | 1.35 |
| Y178 | 2.10 | 0.83 |
| T195 | 2.01 | 0.77 |
| I204 | 1.89 | 0.69 |
| N216 | 1.77 | 0.68 |
| L223 | 3.62 | 0.50 |

**Supplementary File 1n**: *R_2eff_* values measured at different CPMG frequencies from CPMG relaxation dispersion experiment for bound TRBP2-dsRBD2 at 600 MHz NMR spectrometer.

| **Residue Number** | ***R_2ef_f* at CPMG frequency (s^-1^)** | | | | | | | | | | | | | |
| --- | --- | --- | --- | --- | --- | --- | --- | --- | --- | --- | --- | --- | --- | --- |
|  |  | | | | | | | | | | | **Repeat** | | |
|  | **25** | **50** | **75** | **125** | **175** | **275** | **375** | **525** | **675** | **825** | **1000** | **125** | **375** | **825** |
| error | 2.52 | 2.21 | 1.84 | 2.15 | 1.98 | 3.09 | 2.93 | 2.52 | 2.52 | 2.93 | 2.97 | 2.73 | 2.90 | 2.99 |
| S151 | 6.51 | 3.25 | 6.04 | 3.11 | 1.98 | 3.44 | 7.08 | 8.93 | 5.47 | 6.04 | 9.84 | 2.54 | 6.00 | 8.69 |
| N152 | 7.15 | 6.40 | 6.58 | 6.09 | 4.19 | 8.78 | 8.75 | 9.35 | 11.15 | 6.55 | 11.67 | 6.47 | 7.74 | 11.67 |
| A153 | 5.19 | 5.37 | 6.15 | 6.21 | 6.76 | 6.68 | 6.51 | 7.28 | 8.83 | 6.15 | 9.70 | 6.11 | 6.98 | 9.14 |
| Q154 | 2.87 | 2.51 | 2.99 | 3.31 | 6.21 | 4.60 | 4.25 | 5.85 | 6.27 | 2.99 | 7.90 | 3.32 | 4.93 | 7.49 |
| Q155 | 0.96 | 1.65 | 1.59 | 1.30 | 4.47 | 2.14 | 2.30 | 3.45 | 4.79 | 1.59 | 6.20 | 1.56 | 2.44 | 5.42 |
| S156 | 22.20 | 21.30 | 20.80 | 22.50 | 22.00 | 18.60 | 23.50 | 20.20 | 26.50 | 20.80 | 24.40 | 21.60 | 21.80 | 24.80 |
| E157 | 13.70 | 12.60 | 12.20 | 11.20 | 9.84 | 12.10 | 11.40 | 12.10 | 12.70 | 12.40 | 13.80 | 11.20 | 10.90 | 13.30 |
| C158 | 5.30 | 5.51 | 5.94 | 5.51 | 1.87 | 7.03 | 7.48 | 6.13 | 9.75 | 5.93 | 10.90 | 5.72 | 7.944 | 9.75 |
| G162 | 18.00 | 20.60 | 19.90 | 20.00 | 17.60 | 17.40 | 19.80 | 23.70 | 23.20 | 19.90 | 22.30 | 23.10 | 24.00 | 22.40 |
| A163 | 32.50 | 25.00 | 21.20 | 24.20 | 19.50 | 22.00 | 26.10 | 23.80 | 28.20 | 21.20 | 24.40 | 22.00 | 21.60 | 26.40 |
| L164 | 21.87 | 22.89 | 19.81 | 21.48 | 19.63 | 23.48 | 25.89 | 25.21 | 20.26 | 19.81 | 21.48 | 22.07 | 20.54 | 23.90 |
| Q165 | 23.20 | 24.30 | 20.20 | 19.30 | 22.80 | 20.50 | 20.30 | 21.90 | 22.00 | 20.20 | 25.00 | 22.50 | 22.10 | 24.20 |
| E166 | 21.50 | 20.10 | 20.20 | 20.70 | 19.80 | 18.00 | 20.30 | 20.70 | 23.60 | 20.20 | 25.40 | 19.80 | 20.80 | 22.70 |
| L167 | 16.70 | 17.80 | 23.20 | 17.50 | 22.10 | 20.00 | 20.30 | 18.20 | 22.10 | 23.00 | 22.40 | 21.10 | 19.70 | 25.20 |
| V168 | 11.70 | 12.40 | 9.58 | 11.00 | 9.46 | 10.70 | 10.30 | 11.10 | 12.40 | 9.58 | 14.60 | 10.80 | 11.70 | 9.98 |
| V169 | 24.80 | 25.70 | 25.40 | 24.00 | 16.20 | 22.30 | 20.00 | 24.50 | 26.30 | 25.40 | 23.80 | 31.60 | 22.90 | 26.70 |
| Q170 | 23.00 | 21.80 | 22.40 | 21.30 | 22.70 | 23.30 | 21.40 | 21.20 | 21.70 | 22.40 | 25.40 | 20.90 | 22.40 | 23.40 |
| G172 | 19.00 | 17.50 | 16.70 | 18.60 | 18.00 | 19.20 | 15.80 | 18.80 | 20.20 | 16.70 | 20.00 | 15.90 | 16.90 | 20.80 |
| W173 | 17.40 | 17.10 | 16.90 | 18.30 | 19.10 | 18.50 | 19.20 | 19.80 | 17.90 | 17.20 | 19.20 | 16.10 | 17.40 | 18.90 |
| E177 | 16.30 | 17.00 | 19.40 | 15.10 | 16.10 | 18.80 | 21.70 | 16.90 | 18.60 | 19.40 | 19.20 | 19.20 | 17.70 | 21.70 |
| Y178 | 11.60 | 11.70 | 14.90 | 13.70 | 15.60 | 13.60 | 13.60 | 13.20 | 15.40 | 14.90 | 21.40 | 15.80 | 18.10 | 14.00 |
| T179 | 19.60 | 15.30 | 18.70 | 19.50 | 18.40 | 19.00 | 20.20 | 19.00 | 18.00 | 18.70 | 24.00 | 18.70 | 21.70 | 24.80 |
| V180 | 16.60 | 16.60 | 16.60 | 13.80 | 18.10 | 20.90 | 16.60 | 16.30 | 19.50 | 16.60 | 16.10 | 21.10 | 11.70 | 20.20 |
| T181 | 24.10 | 17.50 | 18.90 | 19.00 | 19.20 | 21.80 | 22.80 | 15.10 | 23.60 | 18.90 | 21.80 | 17.10 | 23.60 | 19.00 |
| Q182 | 19.10 | 22.80 | 18.50 | 19.10 | 18.70 | 15.00 | 17.70 | 18.10 | 17.70 | 18.50 | 23.20 | 18.50 | 20.70 | 17.10 |
| E183 | 19.50 | 17.80 | 20.80 | 19.50 | 19.10 | 18.70 | 21.40 | 18.30 | 20.10 | 20.80 | 19.90 | 20.70 | 20.60 | 21.10 |
| S184 | 16.87 | 12.49 | 16.08 | 10.31 | 10.31 | 6.15 | 12.11 | 14.72 | 11.43 | 16.07 | 15.11 | 15.76 | 15.51 | 17.08 |
| G185 | 16.40 | 20.40 | 16.40 | 14.10 | 18.80 | 16.00 | 17.20 | 12.10 | 15.40 | 16.40 | 21.80 | 20.10 | 17.90 | 17.70 |
| A187 | 23.70 | 23.21 | 19.29 | 23.37 | 23.37 | 35.06 | 24.14 | 20.03 | 30.58 | 19.81 | 31.74 | 22.32 | 37.16 | 20.48 |
| R189 | 14.07 | 21.04 | 16.42 | 13.58 | 13.58 | 15.71 | 17.90 | 13.99 | 20.05 | 16.07 | 16.74 | 21.90 | 17.66 | 14.72 |
| E191 | 18.40 | 17.40 | 16.80 | 16.40 | 18.00 | 16.50 | 14.60 | 18.60 | 15.40 | 16.80 | 19.00 | 18.50 | 14.90 | 17.50 |
| T193 | 23.80 | 18.00 | 23.70 | 24.10 | 22.60 | 21.00 | 16.20 | 21.80 | 19.00 | 23.70 | 22.80 | 21.10 | 20.20 | 25.10 |
| M194 | 17.80 | 13.00 | 13.00 | 26.50 | 8.49 | 27.50 | 13.50 | 14.00 | 21.30 | 13.00 | 24.40 | 26.80 | 9.79 | 12.50 |
| T195 | 30.10 | 23.50 | 25.10 | 21.10 | 26.60 | 25.80 | 19.20 | 18.90 | 30.90 | 25.10 | 28.00 | 23.50 | 28.60 | 24.00 |
| C196 | 17.10 | 13.90 | 19.40 | 29.20 | 13.20 | 19.50 | 23.40 | 8.60 | 19.40 | 20.30 | 21.90 | 19.40 | 8.65 | 15.50 |
| R197 | 15.60 | 13.10 | 11.50 | 13.70 | 17.80 | 19.00 | 15.20 | 13.30 | 25.10 | 21.60 | 27.40 | 26.10 | 11.80 | 27.40 |
| V198 | 18.10 | 17.90 | 21.70 | 22.50 | 18.80 | 12.40 | 13.90 | 21.50 | 11.40 | 12.60 | 14.20 | 23.00 | 29.70 | 19.80 |
| E199 | 18.70 | 19.80 | 17.40 | 18.20 | 20.70 | 20.30 | 19.60 | 20.30 | 18.50 | 17.40 | 20.90 | 19.10 | 20.00 | 20.80 |
| R200 | 21.80 | 27.10 | 25.80 | 14.30 | 21.90 | 27.20 | 17.70 | 27.90 | 21.70 | 26.60 | 25.20 | 16.10 | 21.20 | 20.40 |
| F201 | 21.50 | 19.60 | 19.70 | 21.20 | 20.00 | 17.90 | 19.50 | 19.30 | 23.00 | 19.70 | 22.40 | 17.90 | 20.10 | 23.30 |
| I202 | 15.20 | 18.60 | 15.00 | 15.50 | 15.20 | 16.70 | 17.20 | 22.00 | 20.60 | 15.00 | 16.70 | 8.95 | 22.20 | 19.80 |
| E203 | 21.30 | 17.90 | 17.10 | 17.10 | 20.10 | 19.30 | 20.80 | 20.40 | 21.20 | 17.10 | 23.20 | 17.40 | 19.80 | 23.90 |
| I204 | 26.90 | 18.20 | 24.00 | 16.30 | 21.00 | 24.30 | 23.30 | 22.00 | 22.40 | 24.00 | 25.60 | 27.50 | 25.30 | 24.10 |
| G205 | 20.50 | 18.00 | 19.00 | 20.50 | 17.90 | 24.00 | 21.60 | 15.30 | 24.50 | 19.00 | 25.10 | 19.70 | 26.20 | 21.20 |
| S206 | 18.40 | 18.30 | 16.20 | 15.50 | 16.30 | 18.40 | 18.70 | 18.90 | 21.30 | 16.20 | 21.50 | 19.20 | 20.30 | 17.80 |
| G207 | 19.30 | 20.20 | 18.80 | 19.30 | 17.50 | 18.70 | 17.40 | 20.40 | 19.20 | 18.80 | 22.00 | 20.70 | 22.40 | 19.00 |
| T208 | 11.70 | 16.80 | 11.80 | 23.50 | 16.90 | 12.50 | 14.50 | 22.60 | 9.57 | 11.80 | 11.80 | 13.50 | 17.40 | 18.60 |
| L212 | 17.20 | 24.10 | 22.50 | 22.30 | 25.30 | 15.90 | 16.40 | 17.30 | 22.60 | 22.50 | 27.60 | 25.80 | 15.60 | 25.80 |
| A213 | 18.30 | 18.70 | 21.70 | 18.00 | 16.50 | 15.70 | 21.20 | 15.70 | 22.70 | 22.20 | 19.60 | 18.50 | 19.10 | 15.90 |
| R215 | 21.70 | 17.70 | 26.60 | 17.30 | 26.70 | 17.10 | 18.50 | 16.00 | 21.80 | 26.60 | 28.90 | 21.40 | 12.20 | 27.40 |
| N216 | 6.54 | 3.31 | 6.10 | 3.17 | 4.25 | 3.50 | 7.14 | 9.19 | 5.51 | 6.10 | 9.90 | 2.60 | 6.06 | 8.75 |
| A217 | 21.40 | 17.30 | 16.10 | 23.50 | 27.00 | 22.40 | 24.10 | 23.00 | 23.00 | 16.10 | 27.20 | 21.70 | 22.90 | 20.80 |
| A218 | 13.10 | 20.60 | 19.20 | 19.00 | 18.90 | 18.70 | 19.40 | 22.20 | 18.10 | 19.20 | 17.40 | 19.40 | 17.20 | 26.60 |
| A219 | 19.90 | 19.50 | 21.30 | 20.60 | 21.80 | 21.50 | 22.90 | 21.60 | 22.50 | 21.30 | 29.90 | 23.40 | 23.50 | 25.00 |
| M221 | 12.58 | 13.92 | 12.18 | 8.97 | 13.30 | 15.67 | 15.22 | 25.21 | 16.92 | 12.18 | 15.53 | 14.11 | 14.37 | 17.40 |
| L222 | 19.90 | 21.50 | 19.80 | 18.70 | 21.00 | 20.30 | 18.90 | 20.10 | 20.10 | 19.80 | 27.00 | 18.20 | 21.80 | 23.00 |
| R224 | 23.50 | 15.60 | 21.60 | 18.10 | 21.80 | 20.40 | 14.10 | 22.30 | 23.20 | 21.60 | 21.70 | 19.50 | 21.80 | 25.80 |
| L223 | 22.80 | 21.10 | 20.10 | 19.30 | 20.20 | 21.80 | 23.50 | 25.30 | 26.00 | 20.10 | 39.40 | 21.10 | 33.00 | 34.50 |
| V225 | 17.40 | 15.30 | 21.60 | 16.20 | 18.10 | 17.50 | 25.70 | 19.10 | 16.20 | 21.80 | 22.60 | 17.50 | 16.70 | 22.50 |
| T227 | 14.70 | 15.00 | 12.30 | 13.60 | 13.40 | 13.90 | 15.50 | 12.60 | 14.60 | 12.30 | 17.00 | 13.40 | 15.40 | 13.60 |
| V228 | 9.09 | 9.59 | 8.98 | 8.41 | 9.01 | 9.09 | 9.48 | 10.10 | 10.70 | 8.98 | 12.70 | 8.36 | 9.54 | 11.60 |
| L230 | 0.68 | 1.61 | 1.85 | 1.85 | 2.58 | 2.34 | 3.14 | 3.35 | 4.65 | 1.14 | 5.83 | 2.34 | 2.58 | 4.94 |
| A232 | 2.43 | 2.63 | 3.01 | 3.01 | 3.60 | 4.82 | 4.82 | 5.94 | 6.94 | 3.01 | 9.06 | 2.86 | 4.60 | 8.17 |

**Supplementary File 1o**: *R_1ρ_* relaxation rates measured using HSn pulses (n=1, 2, 4, 6, 8) from HARD experiment for RNA-bound TRBP2-dsRBD2 at 600 MHz NMR spectrometer.

| **Residue**  **Number** | ***R_1ρ_* (s^-1^)** | | | | | | | | | |
| --- | --- | --- | --- | --- | --- | --- | --- | --- | --- | --- |
|  | **HS1** | | **HS2** | | **HS4** | | **HS6** | | **HS8** | |
|  | **Value** | **Error** | **Value** | **Error** | **Value** | **Error** | **Value** | **Error** | **Value** | **Error** |
| A153 | 2.45 | 0.11 | 4.29 | 0.30 | 4.62 | 0.18 | 4.73 | 0.28 | 3.58 | 0.11 |
| Q154 | 2.68 | 0.03 | 4.68 | 0.28 | 4.94 | 0.14 | 5.27 | 0.30 | 3.41 | 0.09 |
| Q155 | 2.35 | 0.12 | 4.54 | 0.39 | 4.48 | 0.27 | 5.06 | 0.20 | 2.93 | 0.16 |
| S156 | 2.73 | 0.28 | 5.41 | 0.32 | 5.56 | 0.38 | 6.20 | 0.17 | 4.38 | 0.23 |
| E157 | 3.52 | 0.10 | 5.42 | 0.23 | 6.00 | 0.15 | 6.14 | 0.25 | 4.62 | 0.13 |
| C158 | 2.99 | 0.74 | 6.28 | 0.74 | 5.45 | 0.56 | 5.27 | 0.56 | 4.47 | 0.25 |
| N159 | 3.72 | 0.91 | 7.78 | 1.05 | 7.49 | 1.12 | 6.83 | 1.32 | 7.02 | 0.73 |
| G162 | 5.52 | 0.64 | 7.17 | 0.55 | 10.55 | 1.03 | 10.27 | 0.49 | 8.35 | 0.67 |
| Q165 | 4.61 | 0.22 | 9.11 | 1.27 | 12.24 | 1.89 | 9.34 | 1.48 | 8.33 | 1.12 |
| E166 | 5.04 | 0.08 | 7.73 | 0.16 | 9.16 | 0.45 | 9.67 | 0.51 | 8.88 | 0.31 |
| L167 | 5.93 | 0.04 | 7.80 | 0.25 | 8.47 | 0.19 | 8.43 | 0.39 | 8.74 | 0.13 |
| V168 | 5.34 | 0.77 | 8.95 | 0.46 | 9.29 | 0.81 | 9.78 | 0.58 | 9.73 | 0.50 |
| V169 | 4.53 | 0.76 | 10.95 | 2.28 | 5.62 | 2.43 | 8.88 | 2.08 | 10.24 | 0.97 |
| Q170 | 5.32 | 0.17 | 9.17 | 1.07 | 9.96 | 0.50 | 9.62 | 1.06 | 8.55 | 1.00 |
| G172 | 4.52 | 0.17 | 7.66 | 0.21 | 9.12 | 0.29 | 10.36 | 0.53 | 9.55 | 0.29 |
| W173 | 4.85 | 0.29 | 7.79 | 0.19 | 8.23 | 0.38 | 9.37 | 0.31 | 8.66 | 0.61 |
| E177 | 3.59 | 0.28 | 6.66 | 0.29 | 8.56 | 0.29 | 8.78 | 0.30 | 7.75 | 0.39 |
| Y178 | 4.88 | 0.12 | 5.15 | 0.39 | 6.02 | 0.24 | 6.20 | 0.47 | 4.98 | 0.37 |
| T179 | 4.57 | 0.28 | 6.85 | 0.72 | 7.93 | 0.34 | 8.49 | 0.59 | 7.59 | 0.62 |
| V180 | 5.04 | 0.70 | 7.11 | 0.60 | 8.87 | 0.39 | 9.68 | 0.35 | 8.34 | 0.42 |
| T181 | 4.77 | 1.02 | 7.60 | 1.28 | 7.97 | 0.38 | 10.72 | 0.94 | 7.86 | 0.76 |
| Q182 | 4.78 | 0.19 | 6.02 | 0.25 | 7.32 | 0.26 | 8.07 | 0.43 | 8.15 | 1.22 |
| E183 | 4.23 | 0.17 | 6.58 | 0.13 | 7.93 | 0.52 | 9.48 | 0.41 | 8.03 | 0.41 |
| G185 | 4.20 | 0.42 | 7.33 | 0.59 | 7.93 | 0.31 | 7.82 | 0.41 | 7.52 | 0.07 |
| R189 | 3.01 | 0.30 | 6.19 | 0.73 | 7.10 | 0.62 | 8.28 | 0.69 | 7.09 | 0.29 |
| E191 | 4.53 | 0.05 | 5.86 | 0.32 | 7.91 | 0.54 | 8.25 | 0.23 | 6.99 | 0.30 |
| T193 | 4.78 | 0.19 | 8.12 | 0.68 | 8.69 | 0.68 | 9.54 | 0.61 | 9.16 | 0.54 |
| M194 | 5.75 | 0.46 | 4.95 | 2.17 | 4.54 | 0.79 | 9.66 | 2.35 | 6.67 | 1.91 |
| T195 | 5.53 | 0.28 | 7.36 | 0.45 | 8.92 | 0.23 | 9.22 | 0.50 | 10.38 | 0.52 |
| C196 | 4.12 | 0.96 | 6.61 | 0.82 | 7.87 | 0.95 | 7.39 | 0.77 | 9.97 | 0.94 |
| R197 | 3.37 | 1.01 | 8.05 | 1.54 | 10.52 | 1.42 | 8.28 | 0.30 | 8.51 | 0.67 |
| V198 | 5.68 | 1.05 | 5.97 | 1.41 | 9.38 | 1.48 | 11.55 | 1.59 | 7.68 | 1.36 |
| E199 | 4.52 | 0.18 | 7.68 | 0.20 | 8.30 | 0.29 | 8.48 | 0.36 | 8.16 | 0.12 |
| 200 | 4.27 | 0.52 | 9.13 | 1.56 | 8.82 | 0.70 | 7.76 | 0.48 | 8.73 | 1.00 |
| F201 | 5.03 | 0.12 | 8.21 | 0.73 | 9.68 | 0.11 | 9.98 | 0.17 | 8.59 | 0.24 |
| I202 | 5.35 | 0.34 | 6.76 | 0.79 | 9.12 | 0.87 | 8.26 | 0.70 | 7.22 | 0.51 |
| E203 | 4.32 | 0.11 | 7.00 | 0.33 | 7.90 | 0.37 | 8.19 | 0.27 | 8.46 | 0.13 |
| I204 | 5.17 | 0.15 | 8.06 | 0.71 | 8.92 | 0.52 | 8.63 | 0.69 | 8.78 | 0.33 |
| G205 | 4.24 | 0.37 | 7.66 | 0.67 | 8.34 | 0.57 | 9.79 | 0.38 | 8.71 | 0.78 |
| S206 | 4.59 | 0.10 | 6.61 | 0.35 | 8.13 | 0.22 | 8.90 | 0.14 | 7.85 | 0.16 |
| G207 | 4.53 | 0.12 | 7.51 | 0.40 | 8.30 | 0.15 | 9.52 | 0.25 | 8.73 | 0.29 |
| T208 | 4.35 | 0.51 | 6.20 | 0.98 | 7.47 | 0.85 | 9.98 | 0.72 | 8.05 | 0.59 |
| S209 | 3.69 | 0.89 | 6.11 | 0.96 | 6.73 | 0.25 | 7.06 | 0.17 | 4.79 | 1.26 |
| L212 | 4.91 | 0.50 | 7.60 | 0.51 | 10.10 | 1.63 | 10.33 | 1.17 | 9.31 | 1.06 |
| A213 | 4.17 | 0.43 | 7.24 | 0.67 | 8.28 | 0.65 | 9.23 | 0.71 | 7.89 | 0.70 |
| R215 | 4.97 | 0.97 | 7.98 | 1.13 | 10.78 | 1.19 | 9.56 | 1.69 | 9.36 | 1.12 |
| N216 | 4.56 | 0.19 | 7.88 | 0.63 | 9.82 | 0.16 | 11.03 | 0.42 | 9.44 | 0.56 |
| A218 | 5.93 | 0.22 | 6.79 | 0.93 | 9.53 | 0.27 | 11.19 | 0.49 | 10.61 | 1.04 |
| A219 | 5.63 | 0.63 | 8.24 | 0.59 | 10.28 | 0.22 | 10.26 | 0.54 | 9.15 | 0.41 |
| M221 | 4.02 | 0.37 | 8.04 | 0.77 | 8.79 | 0.78 | 9.02 | 1.18 | 6.86 | 0.56 |
| L223 | 4.53 | 0.32 | 7.39 | 0.36 | 7.90 | 0.31 | 9.71 | 0.80 | 9.38 | 0.22 |
| R224 | 4.50 | 0.36 | 6.61 | 0.73 | 9.11 | 0.44 | 9.80 | 0.29 | 8.96 | 0.87 |
| V225 | 5.51 | 0.54 | 10.74 | 0.96 | 7.06 | 1.11 | 8.82 | 0.89 | 10.48 | 0.44 |
| T227 | 3.86 | 0.29 | 5.99 | 0.65 | 7.00 | 0.26 | 7.24 | 0.31 | 6.81 | 0.40 |
| V228 | 3.02 | 0.30 | 6.23 | 0.37 | 6.32 | 0.18 | 6.30 | 0.19 | 5.06 | 0.22 |
| L230 | 1.66 | 0.03 | 3.69 | 0.36 | 3.90 | 0.11 | 3.84 | 0.31 | 1.85 | 0.05 |
| A232 | 2.09 | 0.09 | 4.69 | 0.37 | 4.64 | 0.12 | 4.49 | 0.28 | 2.68 | 0.13 |

**Supplementary File 1p**: *R_2ρ_* relaxation rates measured using HSn pulses (n=1, 2, 4, 6, 8) from HARD experiment for RNA-bound TRBP2-dsRBD2 at 600 MHz NMR spectrometer.

| **Residue Number** | ***R_2ρ_* (s^-1^)** | | | | | | | | | | | | | | |
| --- | --- | --- | --- | --- | --- | --- | --- | --- | --- | --- | --- | --- | --- | --- | --- |
|  | **HS1** | | | **HS2** | | | **HS4** | | | **HS6** | | | **HS8** | | |
|  | **Value** | **Error** | **Value** | | **Error** | **Value** | | **Error** | **Value** | | **Error** | **Value** | | **Error** |  |
| S151 | 7.52 | 0.88 | 7.36 | | 0.08 | 8.09 | | 0.39 | 9.85 | | 0.93 | 6.44 | | 0.17 |  |
| A153 | 7.20 | 0.93 | 7.03 | | 0.12 | 6.90 | | 0.66 | 9.42 | | 0.20 | 6.89 | | 0.38 |  |
| Q154 | 5.95 | 0.19 | 5.43 | | 0.29 | 5.39 | | 0.12 | 7.74 | | 0.00 | 5.41 | | 0.54 |  |
| Q155 | 4.06 | 0.56 | 4.54 | | 0.50 | 4.69 | | 2.13 | 7.14 | | 2.55 | 4.43 | | 0.96 |  |
| S156 | 11.98 | 1.44 | 10.14 | | 0.77 | 9.29 | | 0.40 | 11.78 | | 0.66 | 8.98 | | 0.10 |  |
| E157 | 11.42 | 0.63 | 11.44 | | 0.90 | 10.55 | | 0.93 | 13.03 | | 0.04 | 11.13 | | 0.32 |  |
| C158 | 10.99 | 0.56 | 7.60 | | 2.88 | 8.80 | | 3.36 | 5.86 | | 1.50 | 5.66 | | 0.04 |  |
| N159 | 22.76 | 1.58 | 24.66 | | 4.86 | 26.77 | | 5.51 | 26.46 | | 6.61 | 25.79 | | 0.72 |  |
| G162 | 22.34 | 0.84 | 22.53 | | 2.55 | 22.89 | | 2.43 | 19.21 | | 6.19 | 18.27 | | 4.48 |  |
| A163 | 25.68 | 5.44 | 20.88 | | 0.59 | 20.24 | | 2.68 | 29.83 | | 10.75 | 23.13 | | 5.24 |  |
| L164 | 22.11 | 1.08 | 21.66 | | 1.43 | 21.37 | | 2.58 | 22.51 | | 5.18 | 19.30 | | 2.13 |  |
| Q165 | 22.98 | 0.15 | 22.62 | | 1.66 | 22.82 | | 1.03 | 22.58 | | 1.74 | 19.23 | | 0.98 |  |
| E166 | 21.15 | 0.97 | 23.39 | | 1.79 | 21.98 | | 0.91 | 21.58 | | 5.12 | 19.53 | | 0.44 |  |
| L167 | 23.56 | 1.19 | 22.80 | | 0.47 | 22.95 | | 1.58 | 25.44 | | 6.71 | 17.64 | | 1.41 |  |
| V168 | 24.93 | 11.77 | 31.39 | | 3.93 | 29.52 | | 3.91 | 28.40 | | 18.94 | 18.46 | | 0.43 |  |
| V169 | 20.39 | 2.98 | 23.93 | | 0.90 | 21.70 | | 0.94 | 17.28 | | 2.39 | 17.25 | | 2.25 |  |
| Q170 | 21.15 | 0.74 | 22.25 | | 0.06 | 21.78 | | 0.45 | 22.11 | | 2.39 | 19.74 | | 1.63 |  |
| G172 | 21.47 | 2.52 | 19.56 | | 2.17 | 19.37 | | 1.05 | 19.66 | | 0.29 | 20.35 | | 0.38 |  |
| W173 | 20.46 | 1.15 | 19.23 | | 1.52 | 19.90 | | 2.37 | 22.82 | | 0.06 | 19.05 | | 0.04 |  |
| R174 | 15.98 | 2.78 | 15.03 | | 0.86 | 18.07 | | 3.56 | 17.64 | | 5.95 | 12.51 | | 1.18 |  |
| E177 | 12.92 | 0.77 | 12.70 | | 0.80 | 13.02 | | 0.65 | 15.59 | | 0.79 | 12.96 | | 0.30 |  |
| Y178 | 20.19 | 0.27 | 16.56 | | 0.74 | 15.63 | | 0.37 | 18.41 | | 3.01 | 17.56 | | 0.94 |  |
| T179 | 18.75 | 1.11 | 19.94 | | 0.53 | 18.82 | | 1.83 | 22.96 | | 4.09 | 18.83 | | 0.36 |  |
| V180 | 22.01 | 3.27 | 21.46 | | 2.48 | 20.08 | | 4.19 | 23.91 | | 0.50 | 26.12 | | 2.81 |  |
| T181 | 21.68 | 1.07 | 18.85 | | 0.86 | 26.22 | | 2.28 | 22.72 | | 0.69 | 18.85 | | 1.54 |  |
| Q182 | 22.61 | 1.38 | 20.11 | | 0.82 | 20.71 | | 0.04 | 22.64 | | 1.13 | 23.06 | | 3.42 |  |
| E183 | 18.22 | 3.48 | 17.77 | | 1.30 | 18.62 | | 2.35 | 22.15 | | 0.40 | 17.84 | | 1.83 |  |
| G185 | 17.93 | 0.46 | 17.18 | | 0.71 | 18.36 | | 2.42 | 18.95 | | 2.71 | 17.48 | | 0.21 |  |
| E191 | 17.60 | 1.33 | 18.25 | | 0.17 | 17.37 | | 1.06 | 27.71 | | 1.83 | 17.71 | | 0.94 |  |
| T193 | 20.75 | 1.00 | 24.53 | | 1.32 | 23.89 | | 1.95 | 19.42 | | 1.43 | 23.31 | | 2.38 |  |
| M194 | 21.64 | 5.16 | 19.92 | | 12.84 | 15.64 | | 2.44 | 21.92 | | 0.14 | 17.78 | | 8.54 |  |
| T195 | 22.00 | 0.70 | 21.89 | | 2.18 | 20.70 | | 0.25 | 20.08 | | 1.54 | 19.74 | | 2.44 |  |
| C196 | 19.54 | 1.76 | 17.44 | | 0.32 | 18.67 | | 0.14 | 15.72 | | 6.59 | 20.17 | | 2.55 |  |
| R197 | 23.88 | 3.14 | 18.10 | | 5.39 | 16.82 | | 0.17 | 14.73 | | 3.25 | 18.00 | | 4.74 |  |
| V198 | 21.00 | 3.24 | 15.94 | | 5.64 | 15.97 | | 2.03 | 12.01 | | 1.39 | 15.78 | | 2.91 |  |
| E199 | 17.92 | 0.09 | 19.06 | | 1.85 | 18.11 | | 1.08 | 17.92 | | 0.24 | 17.62 | | 0.03 |  |
| 200 | 17.91 | 6.24 | 20.25 | | 1.94 | 24.53 | | 1.66 | 16.34 | | 7.13 | 15.38 | | 0.16 |  |
| F201 | 19.22 | 0.12 | 17.94 | | 0.24 | 18.44 | | 1.67 | 18.23 | | 1.48 | 16.84 | | 0.65 |  |
| I202 | 20.00 | 1.61 | 19.45 | | 0.39 | 16.81 | | 1.66 | 20.95 | | 0.46 | 17.96 | | 0.02 |  |
| E203 | 19.80 | 1.40 | 20.52 | | 0.20 | 18.91 | | 1.30 | 17.10 | | 3.39 | 17.46 | | 1.94 |  |
| I204 | 20.08 | 1.11 | 21.68 | | 0.27 | 21.36 | | 1.32 | 22.82 | | 3.02 | 20.50 | | 3.25 |  |
| G205 | 22.26 | 0.40 | 18.03 | | 0.24 | 19.48 | | 0.28 | 18.40 | | 2.92 | 18.65 | | 3.01 |  |
| S206 | 18.69 | 0.50 | 17.92 | | 2.08 | 17.61 | | 0.08 | 18.80 | | 0.02 | 17.81 | | 0.10 |  |
| G207 | 22.45 | 1.73 | 20.50 | | 2.59 | 21.27 | | 1.82 | 21.01 | | 2.15 | 20.08 | | 1.31 |  |
| T208 | 19.50 | 0.64 | 23.86 | | 0.48 | 20.49 | | 0.79 | 17.94 | | 0.23 | 16.75 | | 3.78 |  |
| S209 | 14.26 | 0.66 | 12.31 | | 0.20 | 14.55 | | 1.79 | 9.77 | | 9.13 | 14.36 | | 3.10 |  |
| L212 | 23.28 | 1.15 | 27.70 | | 2.68 | 24.48 | | 0.90 | 23.33 | | 5.02 | 21.71 | | 7.43 |  |
| A213 | 22.66 | 4.90 | 20.19 | | 1.04 | 20.21 | | 0.53 | 15.27 | | 12.49 | 19.65 | | 1.12 |  |
| R215 | 20.61 | 1.26 | 26.71 | | 1.26 | 23.27 | | 4.53 | 18.58 | | 11.00 | 17.22 | | 3.91 |  |
| N216 | 25.37 | 2.54 | 23.62 | | 0.11 | 21.27 | | 2.74 | 24.28 | | 2.86 | 24.34 | | 0.35 |  |
| A218 | 23.38 | 0.51 | 23.58 | | 0.66 | 20.59 | | 6.55 | 23.59 | | 7.26 | 20.66 | | 0.21 |  |
| A219 | 24.02 | 1.34 | 22.84 | | 0.63 | 21.45 | | 1.20 | 24.16 | | 3.88 | 21.11 | | 1.95 |  |
| M221 | 15.46 | 1.37 | 13.39 | | 1.01 | 17.72 | | 3.58 | 16.92 | | 0.88 | 9.83 | | 1.58 |  |
| L223 | 18.21 | 0.54 | 23.29 | | 1.03 | 21.64 | | 0.76 | 24.19 | | 8.45 | 20.25 | | 0.39 |  |
| R224 | 20.78 | 1.38 | 27.11 | | 0.01 | 26.05 | | 2.72 | 21.81 | | 10.88 | 19.37 | | 3.91 |  |
| V225 | 17.02 | 2.64 | 22.54 | | 9.64 | 12.75 | | 3.81 | 12.07 | | 15.39 | 19.89 | | 1.51 |  |
| T227 | 15.66 | 1.96 | 13.51 | | 0.75 | 13.80 | | 0.66 | 14.58 | | 1.57 | 14.46 | | 0.17 |  |
| V228 | 11.44 | 2.07 | 10.40 | | 1.40 | 11.43 | | 1.75 | 13.93 | | 0.57 | 10.89 | | 0.65 |  |
| L230 | 2.51 | 0.23 | 2.28 | | 0.13 | 2.56 | | 0.51 | 5.01 | | 0.36 | 2.66 | | 0.00 |  |
| A232 | 4.35 | 0.21 | 3.89 | | 0.21 | 4.25 | | 0.55 | 6.80 | | 0.24 | 4.39 | | 0.47 |  |

**Supplementary File 1q:** Dynamics parameters extracted from HARD experimental data from geoHARD method for RNA-bound TRBP2-dsRBD2.

| **Residue Number** | ***k_ex_* (Hz)** | | ***p_B_* (%)** |
| --- | --- | --- | --- |
|  | **Value** | **Error** |  |
| N159 | 109.84 | 6.09 | 8.18 |
| G162 | 33694.84 | 4498.63 | 14.58 |
| Q165 | 144.71 | 39.88 | 3.07 |
| E166 | 117.25 | 18.81 | 3.31 |
| L167 | 33136.76 | 1028.77 | 13.75 |
| V168 | 31361.87 | 381.57 | 23.84 |
| V169 | 157.41 | 44.83 | 3.44 |
| Q170 | 116.96 | 11.92 | 2.92 |
| G172 | 175.47 | 86.44 | 2.47 |
| W173 | 114.07 | 8.10 | 3.51 |
| T179 | 109.72 | 7.08 | 4.01 |
| V180 | 143.37 | 29.57 | 5.40 |
| T181 | 114.44 | 6.67 | 4.89 |
| Q182 | 137.39 | 19.91 | 4.48 |
| E183 | 119.61 | 13.26 | 4.15 |
| G185 | 130.95 | 25.10 | 2.92 |
| E191 | 110.37 | 8.93 | 4.66 |
| T193 | 111.15 | 9.42 | 3.73 |
| C196 | 117.34 | 15.76 | 3.48 |
| R197 | 38548.93 | 3434.10 | 6.46 |
| V198 | 73599.49 | 5723.40 | 27.41 |
| E199 | 114.28 | 11.25 | 2.60 |
| R200 | 34143.21 | 678.61 | 19.36 |
| F201 | 171.94 | 65.01 | 1.11 |
| E203 | 34467.84 | 1310.56 | 21.48 |
| I204 | 111.56 | 15.16 | 3.32 |
| G205 | 154.09 | 34.56 | 2.51 |
| S206 | 120.58 | 16.23 | 3.03 |
| G207 | 29981.15 | 4358.72 | 4.63 |
| T208 | 148.23 | 48.27 | 3.66 |
| S209 | 125.06 | 16.57 | 1.77 |
| L212 | 120.49 | 18.57 | 4.42 |
| A213 | 27995.83 | 4031.29 | 14.63 |
| R215 | 136.63 | 33.31 | 2.96 |
| N216 | 111.11 | 5.78 | 5.34 |
| A219 | 34874.65 | 4100.95 | 15.65 |
| M221 | 61676.83 | 1371.30 | 24.59 |
| L223 | 101.90 | 2.14 | 3.50 |
| R224 | 109.67 | 9.36 | 5.47 |
| V225 | 125.89 | 18.09 | 1.15 |

**Supplementary File 1r**: *R_1_* relaxation rates measured from HARD experiment for apo TRBP2-dsRBD2 at 600 MHz NMR spectrometer.

| **Residue Number** | ***R_1_* (s^-1^)** | |
| --- | --- | --- |
|  | **Value** | **Error** |
| S151 | 1.50 | 0.04 |
| Q154 | 1.57 | 0.07 |
| Q155 | 1.41 | 0.06 |
| S156 | 1.60 | 0.05 |
| E157 | 1.65 | 0.04 |
| N159 | 1.54 | 0.11 |
| G162 | 1.54 | 0.05 |
| A163 | 1.49 | 0.08 |
| Q165 | 1.50 | 0.05 |
| E166 | 1.49 | 0.05 |
| L167 | 1.44 | 0.07 |
| V168 | 1.42 | 0.08 |
| V169 | 1.51 | 0.05 |
| Q170 | 1.48 | 0.06 |
| G172 | 1.50 | 0.06 |
| W173 | 1.50 | 0.06 |
| E177 | 1.46 | 0.06 |
| Y178 | 1.55 | 0.05 |
| T179 | 1.56 | 0.04 |
| V180 | 1.71 | 0.08 |
| T181 | 1.62 | 0.04 |
| Q182 | 1.63 | 0.05 |
| E183 | 1.57 | 0.07 |
| G185 | 1.57 | 0.05 |
| R189 | 1.72 | 0.07 |
| E191 | 1.46 | 0.05 |
| T193 | 1.49 | 0.04 |
| M194 | 1.58 | 0.07 |
| T195 | 1.57 | 0.04 |
| C196 | 1.62 | 0.04 |
| R197 | 1.63 | 0.09 |
| V198 | 1.55 | 0.08 |
| E199 | 1.56 | 0.06 |
| R200 | 1.44 | 0.05 |
| F201 | 1.60 | 0.05 |
| I202 | 1.52 | 0.07 |
| E203 | 1.61 | 0.04 |
| I204 | 1.47 | 0.07 |
| G205 | 1.62 | 0.04 |
| S206 | 1.50 | 0.04 |
| G207 | 1.55 | 0.04 |
| T208 | 1.71 | 0.04 |
| S209 | 1.35 | 0.05 |
| L212 | 1.42 | 0.04 |
| A213 | 1.53 | 0.06 |
| R215 | 1.42 | 0.08 |
| N216 | 1.44 | 0.06 |
| A217 | 1.53 | 0.09 |
| A218 | 1.45 | 0.06 |
| A219 | 1.42 | 0.05 |
| L223 | 1.35 | 0.06 |
| R224 | 1.45 | 0.08 |
| V225 | 1.33 | 0.10 |
| T227 | 1.63 | 0.05 |
| V228 | 1.67 | 0.07 |
| L230 | 1.06 | 0.06 |
| A232 | 1.47 | 0.06 |

**Supplementary File 1s**: *R_1_* relaxation rates measured from HARD experiment for RNA-bound TRBP2-dsRBD2 at 600 MHz NMR spectrometer.

| **Residue Number** | ***R_1_* (s^-1^)** | |
| --- | --- | --- |
|  | **Value** | **Error** |
| S151 | 1.36 | 0.10 |
| A153 | 1.48 | 0.02 |
| Q154 | 1.47 | 0.03 |
| Q155 | 1.17 | 0.04 |
| S156 | 1.41 | 0.13 |
| E157 | 1.55 | 0.11 |
| C158 | 1.51 | 0.17 |
| N159 | 1.67 | 0.24 |
| G162 | 1.21 | 0.21 |
| A163 | 1.28 | 0.11 |
| Q165 | 1.30 | 0.15 |
| E166 | 1.35 | 0.10 |
| L167 | 1.15 | 0.18 |
| V168 | 1.36 | 0.30 |
| V169 | 1.40 | 0.12 |
| Q170 | 1.29 | 0.05 |
| G172 | 1.35 | 0.05 |
| W173 | 1.33 | 0.11 |
| E177 | 1.26 | 0.08 |
| Y178 | 1.43 | 0.08 |
| T179 | 1.39 | 0.09 |
| V180 | 1.50 | 0.23 |
| T181 | 1.46 | 0.13 |
| Q182 | 1.56 | 0.08 |
| E183 | 1.41 | 0.10 |
| G185 | 1.46 | 0.14 |
| R189 | 1.51 | 0.22 |
| E191 | 1.41 | 0.07 |
| T193 | 1.44 | 0.18 |
| M194 | 1.37 | 0.42 |
| T195 | 1.34 | 0.18 |
| C196 | 1.61 | 0.09 |
| R197 | 1.36 | 0.19 |
| V198 | 1.54 | 0.38 |
| E199 | 1.46 | 0.08 |
| R200 | 0.96 | 0.23 |
| F201 | 1.45 | 0.04 |
| I202 | 1.35 | 0.10 |
| E203 | 1.52 | 0.06 |
| I204 | 1.22 | 0.11 |
| G205 | 1.52 | 0.07 |
| S206 | 1.38 | 0.03 |
| G207 | 1.35 | 0.04 |
| T208 | 1.51 | 0.15 |
| S209 | 1.25 | 0.10 |
| L212 | 1.35 | 0.20 |
| A213 | 1.14 | 0.19 |
| R215 | 1.07 | 0.12 |
| N216 | 1.37 | 0.07 |
| A218 | 1.36 | 0.16 |
| A219 | 1.25 | 0.10 |
| M221 | 1.27 | 0.15 |
| L223 | 1.31 | 0.14 |
| R224 | 1.40 | 0.09 |
| V225 | 1.27 | 0.33 |
| T227 | 1.56 | 0.10 |
| V228 | 1.57 | 0.06 |
| L230 | 0.96 | 0.02 |

**Supplementary File 1t**: Assignment Report of TRBP2-dsRBD2 (as obtained from CARA in NMR-STAR 3.1 format)

data_starch_output

###################################################################

# Chemical Shift Ambiguity Index Value Definitions #

# #

# The values other than 1 are used for those atoms with different #

# chemical shifts that cannot be assigned to stereospecific atoms #

# or to specific residues or chains. #

# #

# Index Value Definition #

# #

# 1 Unique (including isolated methyl protons, #

# geminal atoms, and geminal methyl #

# groups with identical chemical shifts) #

# (e.g. ILE HD11, HD12, HD13 protons) #

# 2 Ambiguity of geminal atoms or geminal methyl #

# proton groups (e.g. ASP HB2 and HB3 #

# protons, LEU CD1 and CD2 carbons, or #

# LEU HD11, HD12, HD13 and HD21, HD22, #

# HD23 methyl protons) #

# 3 Aromatic atoms on opposite sides of #

# symmetrical rings (e.g. TYR HE1 and HE2 #

# protons) #

# 4 Intraresidue ambiguities (e.g. LYS HG and #

# HD protons or TRP HZ2 and HZ3 protons) #

# 5 Interresidue ambiguities (LYS 12 vs. LYS 27) #

# 6 Intermolecular ambiguities (e.g. ASP 31 CA #

# in monomer 1 and ASP 31 CA in monomer 2 #

# of an asymmetrical homodimer, duplex #

# DNA assignments, or other assignments #

# that may apply to atoms in one or more #

# molecule in the molecular assembly) #

# 9 Ambiguous, specific ambiguity not defined #

# #

###################################################################

loop_

_Atom_chem_shift.Atom_ID

_Atom_chem_shift.Comp_index_ID

_Atom_chem_shift.Comp_ID

_Atom_chem_shift.Atom_ID

_Atom_chem_shift.Atom_type

_Atom_chem_shift.Val

_Atom_chem_shift.Val_err

_Atom_chem_shift.Ambiguity_code

C 1 SER C C 171.651 0.3 1

CA 1 SER CA C 59.107 0.3 1

CB 1 SER CB C 66.892 0.3 1

H 1 SER H H 8.050 0.020 1

HA 1 SER HA H 4.658 0.020 1

HB2 1 SER HB2 H 4.128 0.020 1

HB3 1 SER HB3 H 4.128 0.020 1

N 1 SER N N 116.264 0.3 1

C 2 ASN C C 173.474 0.3 1

CA 2 ASN CA C 53.594 0.3 1

CB 2 ASN CB C 26.344 0.3 1

H 2 ASN H H 8.316 0.020 1

HA 2 ASN HA H 4.660 0.020 1

HB2 2 ASN HB2 H 2.282 0.020 1

HB3 2 ASN HB3 H 2.282 0.020 2

N 2 ASN N N 122.883 0.3 1

C 3 ALA C C 174.311 0.3 1

CA 3 ALA CA C 60.321 0.3 1

CB 3 ALA CB C 29.395 0.3 1

H 3 ALA H H 8.289 0.020 1

HA 3 ALA HA H 4.188 0.020 1

HB 3 ALA HB H 1.548 0.020 1

N 3 ALA N N 122.526 0.3 1

C 4 GLN C C 172.965 0.3 1

CA 4 GLN CA C 51.009 0.3 1

CB 4 GLN CB C 38.197 0.3 1

H 4 GLN H H 8.212 0.020 1

HA 4 GLN HA H 4.700 0.020 1

HB2 4 GLN HB2 H 1.669 0.020 1

HB3 4 GLN HB3 H 1.577 0.020 2

HG2 4 GLN HG2 H 2.586 0.020 1

HG3 4 GLN HG3 H 2.817 0.020 2

N 4 GLN N N 119.927 0.3 1

C 5 GLN C C 173.494 0.3 1

CA 5 GLN CA C 53.100 0.3 1

CB 5 GLN CB C 26.290 0.3 1

H 5 GLN H H 8.214 0.020 1

HA 5 GLN HA H 4.687 0.020 1

HB2 5 GLN HB2 H 1.585 0.020 1

HB3 5 GLN HB3 H 1.585 0.020 2

HG2 5 GLN HG2 H 2.551 0.020 1

HG3 5 GLN HG3 H 2.831 0.020 2

N 5 GLN N N 120.433 0.3 1

C 6 SER C C 171.706 0.3 1

CA 6 SER CA C 56.270 0.3 1

CB 6 SER CB C 60.665 0.3 1

H 6 SER H H 8.241 0.020 1

HA 6 SER HA H 4.690 0.020 1

HB2 6 SER HB2 H 3.730 0.020 1

HB3 6 SER HB3 H 4.196 0.020 2

N 6 SER N N 116.723 0.3 1

C 7 GLU C C 173.718 0.3 1

CA 7 GLU CA C 53.829 0.3 1

CB 7 GLU CB C 27.232 0.3 1

H 7 GLU H H 8.197 0.020 1

HA 7 GLU HA H 4.676 0.020 1

HB2 7 GLU HB2 H 1.905 0.020 1

HB3 7 GLU HB3 H 1.905 0.020 1

HG2 7 GLU HG2 H 2.083 0.020 1

HG3 7 GLU HG3 H 2.083 0.020 1

N 7 GLU N N 121.958 0.3 1

CA 8 CYS CA C 50.998 0.3 1

CB 8 CYS CB C 38.589 0.3 1

H 8 CYS H H 8.291 0.020 1

HA 8 CYS HA H 4.550 0.020 1

HB2 8 CYS HB2 H 2.578 0.020 1

HB3 8 CYS HB3 H 2.578 0.020 1

N 8 CYS N N 120.809 0.3 1

C 9 ASN C C 170.017 0.3 1

CA 9 ASN CA C 47.594 0.3 1

CB 9 ASN CB C 35.659 0.3 1

H 9 ASN H H 8.703 0.020 1

HA 9 ASN HA H 4.679 0.020 1

HB2 9 ASN HB2 H 2.704 0.020 2

HB3 9 ASN HB3 H 2.991 0.020 2

N 9 ASN N N 120.150 0.3 1

C 11 VAL C C 177.012 0.3 1

CA 11 VAL CA C 63.640 0.3 1

CB 11 VAL CB C 28.907 0.3 1

H 11 VAL H H 7.731 0.020 1

HA 11 VAL HA H 4.516 0.020 1

HB 11 VAL HB H 3.104 0.020 1

HG1 11 VAL HG1 H 1.968 0.020 1

HG2 11 VAL HG2 H 1.968 0.020 1

N 11 VAL N N 117.949 0.3 1

C 12 GLY C C 173.359 0.3 1

CA 12 GLY CA C 44.424 0.3 1

H 12 GLY H H 7.285 0.020 1

HA2 12 GLY HA2 H 3.763 0.020 1

HA3 12 GLY HA3 H 3.763 0.020 1

N 12 GLY N N 108.233 0.3 1

C 13 ALA C C 178.136 0.3 1

CA 13 ALA CA C 52.023 0.3 1

CB 13 ALA CB C 15.946 0.3 1

H 13 ALA H H 8.273 0.020 1

HA 13 ALA HA H 4.023 0.020 1

HB 13 ALA HB H 1.339 0.020 1

N 13 ALA N N 124.528 0.3 1

C 14 LEU C C 178.136 0.3 1

CA 14 LEU CA C 51.916 0.3 1

CB 14 LEU CB C 15.823 0.3 1

H 14 LEU H H 8.436 0.020 1

HA 14 LEU HA H 4.016 0.020 1

HB2 14 LEU HB2 H 2.000 0.020 1

HB3 14 LEU HB3 H 2.000 0.020 1

N 14 LEU N N 119.386 0.3 1

C 15 GLN C C 174.032 0.3 1

CA 15 GLN CA C 57.538 0.3 1

CB 15 GLN CB C 25.246 0.3 1

H 15 GLN H H 7.540 0.020 1

HA 15 GLN HA H 3.634 0.020 1

HB2 15 GLN HB2 H 2.154 0.020 1

HB3 15 GLN HB3 H 2.154 0.020 1

HG2 15 GLN HG2 H 2.397 0.020 1

HG3 15 GLN HG3 H 2.397 0.020 1

N 15 GLN N N 118.263 0.3 1

C 16 GLU C C 176.016 0.3 1

CA 16 GLU CA C 56.389 0.3 1

CB 16 GLU CB C 26.832 0.3 1

H 16 GLU H H 7.775 0.020 1

HA 16 GLU HA H 3.866 0.020 1

HB2 16 GLU HB2 H 1.967 0.020 1

HB3 16 GLU HB3 H 1.967 0.020 1

N 16 GLU N N 115.882 0.3 1

CA 17 LEU CA C 56.442 0.3 1

H 17 LEU H H 7.622 0.020 1

HA 17 LEU HA H 3.946 0.020 1

HB2 17 LEU HB2 H 1.726 0.020 1

HB3 17 LEU HB3 H 1.726 0.020 1

N 17 LEU N N 120.591 0.3 1

C 18 VAL C C 175.377 0.3 1

CA 18 VAL CA C 63.745 0.3 1

CB 18 VAL CB C 28.297 0.3 1

H 18 VAL H H 8.318 0.020 1

HA 18 VAL HA H 3.397 0.020 1

HB 18 VAL HB H 2.322 0.020 1

N 18 VAL N N 115.936 0.3 1

C 19 VAL C C 177.933 0.3 1

CA 19 VAL CA C 63.494 0.3 1

CB 19 VAL CB C 28.541 0.3 1

H 19 VAL H H 7.863 0.020 1

HA 19 VAL HA H 3.762 0.020 1

HB 19 VAL HB H 2.055 0.020 1

HG1 19 VAL HG1 H 0.911 0.020 2

HG2 19 VAL HG2 H 0.911 0.020 1

N 19 VAL N N 119.974 0.3 1

C 20 GLN C C 175.579 0.3 1

CA 20 GLN CA C 55.967 0.3 1

CB 20 GLN CB C 25.238 0.3 1

H 20 GLN H H 7.766 0.020 1

HA 20 GLN HA H 3.883 0.020 1

HB2 20 GLN HB2 H 2.198 0.020 1

HB3 20 GLN HB3 H 2.198 0.020 1

HG2 20 GLN HG2 H 2.389 0.020 1

HG3 20 GLN HG3 H 2.389 0.020 1

N 20 GLN N N 121.355 0.3 1

C 22 GLY C C 172.249 0.3 1

CA 22 GLY CA C 42.640 0.3 1

H 22 GLY H H 7.670 0.020 1

HA2 22 GLY HA2 H 3.988 0.020 1

HA3 22 GLY HA3 H 3.988 0.020 2

N 22 GLY N N 106.857 0.3 1

C 23 TRP C C 172.485 0.3 1

CA 23 TRP CA C 50.567 0.3 1

CB 23 TRP CB C 28.907 0.3 1

H 23 TRP H H 7.536 0.020 1

HA 23 TRP HA H 4.968 0.020 1

HB2 23 TRP HB2 H 3.277 0.020 1

HB3 23 TRP HB3 H 3.223 0.020 2

HD1 23 TRP HD1 H 7.195 0.020 1

HE1 23 TRP HE1 H 9.950 0.020 1

HZ2 23 TRP HZ2 H 6.757 0.020 1

N 23 TRP N N 121.648 0.3 1

C 24 ARG C C 173.124 0.3 1

CA 24 ARG CA C 53.780 0.3 1

CB 24 ARG CB C 27.809 0.3 1

H 24 ARG H H 8.401 0.020 1

HA 24 ARG HA H 4.056 0.020 1

HB2 24 ARG HB2 H 1.761 0.020 1

HB3 24 ARG HB3 H 1.761 0.020 1

HE 24 ARG HE H 3.174 0.020 1

N 24 ARG N N 119.407 0.3 1

CA 25 LEU CA C 53.728 0.3 1

CB 25 LEU CB C 27.813 0.3 1

H 25 LEU H H 7.786 0.020 1

HA 25 LEU HA H 4.222 0.020 1

N 25 LEU N N 118.119 0.3 1

C 27 GLU C C 172.956 0.3 1

CA 27 GLU CA C 51.604 0.3 1

CB 27 GLU CB C 29.883 0.3 1

H 27 GLU H H 8.353 0.020 1

HA 27 GLU HA H 4.674 0.020 1

HB2 27 GLU HB2 H 1.936 0.020 1

HB3 27 GLU HB3 H 2.181 0.020 2

HG2 27 GLU HG2 H 3.006 0.020 1

HG3 27 GLU HG3 H 3.006 0.020 2

N 27 GLU N N 122.417 0.3 1

CA 28 TYR CA C 51.602 0.3 1

H 28 TYR H H 9.065 0.020 1

HA 28 TYR HA H 5.594 0.020 1

HB2 28 TYR HB2 H 2.720 0.020 1

HB3 28 TYR HB3 H 2.720 0.020 2

HD1 28 TYR HD1 H 6.900 0.020 1

HE1 28 TYR HE1 H 6.545 0.020 1

N 28 TYR N N 126.987 0.3 1

C 29 THR C C 170.601 0.3 1

CA 29 THR CA C 58.160 0.3 1

CB 29 THR CB C 68.776 0.3 1

H 29 THR H H 8.531 0.020 1

HA 29 THR HA H 4.540 0.020 1

HB 29 THR HB H 3.840 0.020 1

HG2 29 THR HG2 H 1.073 0.020 1

N 29 THR N N 117.650 0.3 1

C 30 VAL C C 173.258 0.3 1

CA 30 VAL CA C 60.094 0.3 1

CB 30 VAL CB C 29.319 0.3 1

H 30 VAL H H 8.990 0.020 1

HA 30 VAL HA H 4.595 0.020 1

HB 30 VAL HB H 2.116 0.020 1

HG1 30 VAL HG1 H 1.068 0.020 1

HG2 30 VAL HG2 H 1.068 0.020 1

N 30 VAL N N 127.369 0.3 1

C 31 THR C C 172.047 0.3 1

CA 31 THR CA C 59.394 0.3 1

CB 31 THR CB C 66.291 0.3 1

H 31 THR H H 8.705 0.020 1

HA 31 THR HA H 4.349 0.020 1

HB 31 THR HB H 4.074 0.020 1

HG1 31 THR HG1 H 1.087 0.020 1

N 31 THR N N 121.646 0.3 1

C 32 GLN C C 170.904 0.3 1

CA 32 GLN CA C 53.471 0.3 1

CB 32 GLN CB C 28.907 0.3 1

H 32 GLN H H 7.885 0.020 1

HA 32 GLN HA H 4.685 0.020 1

HB2 32 GLN HB2 H 1.935 0.020 1

HB3 32 GLN HB3 H 1.935 0.020 1

HG2 32 GLN HG2 H 2.201 0.020 1

HG3 32 GLN HG3 H 2.201 0.020 1

HE21 32 GLN HE21 H 7.261 0.020 1

HE22 32 GLN HE22 H 6.888 0.020 1

N 32 GLN N N 121.190 0.3 1

C 33 GLU C C 172.417 0.3 1

CA 33 GLU CA C 52.370 0.3 1

CB 33 GLU CB C 28.769 0.3 1

H 33 GLU H H 8.316 0.020 1

HA 33 GLU HA H 4.685 0.020 1

HB2 33 GLU HB2 H 1.781 0.020 1

N 33 GLU N N 124.085 0.3 1

C 34 SER C C 170.904 0.3 1

CA 34 SER CA C 54.725 0.3 1

CB 34 SER CB C 62.221 0.3 1

H 34 SER H H 8.197 0.020 1

HA 34 SER HA H 4.687 0.020 1

HB2 34 SER HB2 H 3.635 0.020 1

HB3 34 SER HB3 H 3.635 0.020 1

HG 34 SER HG H 2.603 0.020 1

N 34 SER N N 118.212 0.3 1

C 35 GLY C C 169.424 0.3 1

CA 35 GLY CA C 41.480 0.3 1

H 35 GLY H H 8.147 0.020 1

HA2 35 GLY HA2 H 3.837 0.020 2

HA3 35 GLY HA3 H 4.692 0.020 2

N 35 GLY N N 109.048 0.3 1

H 37 ALA H H 8.246 0.020 1

HA 37 ALA HA H 4.254 0.020 1

HB 37 ALA HB H 1.673 0.020 1

N 37 ALA N N 120.862 0.3 1

C 39 ARG C C 171.442 0.3 1

CA 39 ARG CA C 52.850 0.3 1

CB 39 ARG CB C 27.564 0.3 1

H 39 ARG H H 7.719 0.020 1

HA 39 ARG HA H 4.660 0.020 1

HB2 39 ARG HB2 H 1.568 0.020 1

HB3 39 ARG HB3 H 1.568 0.020 1

HG2 39 ARG HG2 H 1.353 0.020 1

HG3 39 ARG HG3 H 1.353 0.020 1

HD2 39 ARG HD2 H 2.093 0.020 1

HD3 39 ARG HD3 H 2.093 0.020 1

HE 39 ARG HE H 3.053 0.020 1

N 39 ARG N N 123.947 0.3 1

C 41 GLU C C 172.384 0.3 1

CA 41 GLU CA C 52.842 0.3 1

CB 41 GLU CB C 29.395 0.3 1

H 41 GLU H H 8.128 0.020 1

HA 41 GLU HA H 4.367 0.020 1

HB2 41 GLU HB2 H 1.943 0.020 1

HB3 41 GLU HB3 H 1.943 0.020 1

HG2 41 GLU HG2 H 1.642 0.020 1

HG3 41 GLU HG3 H 1.642 0.020 1

N 41 GLU N N 119.493 0.3 1

C 42 PHE C C 172.384 0.3 1

CA 42 PHE CA C 52.842 0.3 1

CB 42 PHE CB C 29.395 0.3 1

H 42 PHE H H 8.775 0.020 1

HA 42 PHE HA H 4.880 0.020 1

HB2 42 PHE HB2 H 2.553 0.020 1

HB3 42 PHE HB3 H 2.553 0.020 1

HD1 42 PHE HD1 H 6.881 0.020 1

HE1 42 PHE HE1 H 7.236 0.020 1

N 42 PHE N N 121.600 0.3 1

C 43 THR C C 170.803 0.3 1

CA 43 THR CA C 58.772 0.3 1

CB 43 THR CB C 67.826 0.3 1

H 43 THR H H 8.717 0.020 1

HA 43 THR HA H 5.105 0.020 1

HB 43 THR HB H 3.836 0.020 1

HG2 43 THR HG2 H 1.068 0.020 1

N 43 THR N N 116.346 0.3 1

CA 44 MET CA C 58.782 0.3 1

H 44 MET H H 9.360 0.020 1

HA 44 MET HA H 5.392 0.020 1

HB2 44 MET HB2 H 1.896 0.020 1

HB3 44 MET HB3 H 1.896 0.020 2

HG2 44 MET HG2 H 2.327 0.020 1

HG3 44 MET HG3 H 2.327 0.020 1

N 44 MET N N 125.128 0.3 1

C 45 THR C C 170.904 0.3 1

CA 45 THR CA C 58.162 0.3 1

CB 45 THR CB C 67.634 0.3 1

H 45 THR H H 9.190 0.020 1

HA 45 THR HA H 4.937 0.020 1

HB 45 THR HB H 3.910 0.020 1

HG1 45 THR HG1 H 1.032 0.020 1

N 45 THR N N 116.395 0.3 1

C 46 CYS C C 169.350 0.3 1

CA 46 CYS CA C 54.079 0.3 1

CB 46 CYS CB C 26.896 0.3 1

H 46 CYS H H 9.124 0.020 1

HA 46 CYS HA H 4.566 0.020 1

HB2 46 CYS HB2 H 2.260 0.020 1

HB3 46 CYS HB3 H 2.260 0.020 1

N 46 CYS N N 124.254 0.3 1

C 47 ARG C C 173.314 0.3 1

CA 47 ARG CA C 51.588 0.3 1

CB 47 ARG CB C 30.648 0.3 1

H 47 ARG H H 8.671 0.020 1

HA 47 ARG HA H 5.201 0.020 1

HB2 47 ARG HB2 H 1.416 0.020 1

HB3 47 ARG HB3 H 1.416 0.020 1

HG2 47 ARG HG2 H 1.232 0.020 1

HG3 47 ARG HG3 H 1.232 0.020 1

HD2 47 ARG HD2 H 1.658 0.020 1

HD3 47 ARG HD3 H 1.658 0.020 1

N 47 ARG N N 128.627 0.3 1

C 48 VAL C C 170.805 0.3 1

CA 48 VAL CA C 58.397 0.3 1

CB 48 VAL CB C 32.934 0.3 1

H 48 VAL H H 8.096 0.020 1

HA 48 VAL HA H 4.080 0.020 1

HB 48 VAL HB H 2.224 0.020 1

HG1 48 VAL HG1 H 0.975 0.020 1

HG2 48 VAL HG2 H 0.975 0.020 1

N 48 VAL N N 126.933 0.3 1

C 49 GLU C C 173.140 0.3 1

CA 49 GLU CA C 55.357 0.3 1

CB 49 GLU CB C 23.782 0.3 1

H 49 GLU H H 8.859 0.020 1

HA 49 GLU HA H 3.259 0.020 1

HB2 49 GLU HB2 H 1.237 0.020 1

HB3 49 GLU HB3 H 1.237 0.020 1

HG2 49 GLU HG2 H 1.493 0.020 1

HG3 49 GLU HG3 H 1.493 0.020 1

N 49 GLU N N 123.705 0.3 1

C 50 ARG C C 173.133 0.3 1

CA 50 ARG CA C 53.146 0.3 1

CB 50 ARG CB C 26.588 0.3 1

H 50 ARG H H 7.312 0.020 1

HA 50 ARG HA H 4.135 0.020 1

HB2 50 ARG HB2 H 1.300 0.020 1

HG2 50 ARG HG2 H 1.011 0.020 1

N 50 ARG N N 120.474 0.3 1

C 51 PHE C C 173.133 0.3 1

CA 51 PHE CA C 53.146 0.3 1

H 51 PHE H H 8.628 0.020 1

HA 51 PHE HA H 4.660 0.020 1

HB2 51 PHE HB2 H 2.811 0.020 1

HB3 51 PHE HB3 H 3.153 0.020 2

HD1 51 PHE HD1 H 7.068 0.020 1

N 51 PHE N N 122.221 0.3 1

CA 52 ILE CA C 53.634 0.3 1

H 52 ILE H H 8.452 0.020 1

HA 52 ILE HA H 4.943 0.020 1

HB 52 ILE HB H 0.701 0.020 1

HG12 52 ILE HG12 H 1.545 0.020 1

HG13 52 ILE HG13 H 1.545 0.020 1

N 52 ILE N N 121.877 0.3 1

C 53 GLU C C 171.777 0.3 1

CA 53 GLU CA C 51.238 0.3 1

CB 53 GLU CB C 32.080 0.3 1

H 53 GLU H H 8.561 0.020 1

HA 53 GLU HA H 4.697 0.020 1

HB2 53 GLU HB2 H 1.365 0.020 1

HB3 53 GLU HB3 H 1.365 0.020 1

HG2 53 GLU HG2 H 2.132 0.020 1

HG3 53 GLU HG3 H 2.132 0.020 1

N 53 GLU N N 124.418 0.3 1

CB 54 ILE CB C 32.080 0.3 1

H 54 ILE H H 8.845 0.020 1

HA 54 ILE HA H 4.898 0.020 1

HB 54 ILE HB H 0.738 0.020 1

HG2 54 ILE HG2 H 1.545 0.020 1

HD1 54 ILE HD1 H 0.940 0.020 1

N 54 ILE N N 120.479 0.3 1

C 55 GLY C C 169.020 0.3 1

CA 55 GLY CA C 40.943 0.3 1

H 55 GLY H H 9.177 0.020 1

HA2 55 GLY HA2 H 3.672 0.020 1

HA3 55 GLY HA3 H 4.678 0.020 2

N 55 GLY N N 112.301 0.3 1

C 56 SER C C 171.341 0.3 1

CA 56 SER CA C 53.780 0.3 1

CB 56 SER CB C 63.810 0.3 1

H 56 SER H H 8.625 0.020 1

HA 56 SER HA H 5.910 0.020 1

HG 56 SER HG H 3.727 0.020 1

N 56 SER N N 114.811 0.3 1

C 57 GLY C C 169.928 0.3 1

CA 57 GLY CA C 42.860 0.3 1

H 57 GLY H H 8.552 0.020 1

HA2 57 GLY HA2 H 3.948 0.020 1

HA3 57 GLY HA3 H 4.440 0.020 2

N 57 GLY N N 106.946 0.3 1

C 58 THR C C 170.467 0.3 1

CA 58 THR CA C 59.902 0.3 1

CB 58 THR CB C 66.004 0.3 1

H 58 THR H H 8.273 0.020 1

HA 58 THR HA H 4.638 0.020 1

N 58 THR N N 109.376 0.3 1

CA 59 SER CA C 39.410 0.3 1

CB 59 SER CB C 22.842 0.3 1

H 59 SER H H 7.322 0.020 1

HA 59 SER HA H 4.699 0.020 1

HB2 59 SER HB2 H 2.920 0.020 1

HB3 59 SER HB3 H 2.920 0.020 1

N 59 SER N N 112.329 0.3 1

C 60 LYS C C 172.956 0.3 1

CB 60 LYS CB C 28.348 0.3 1

H 60 LYS H H 8.408 0.020 1

HA 60 LYS HA H 4.695 0.020 1

HB2 60 LYS HB2 H 1.948 0.020 1

HB3 60 LYS HB3 H 1.948 0.020 1

N 60 LYS N N 123.979 0.3 1

C 62 LEU C C 172.014 0.3 1

CA 62 LEU CA C 52.846 0.3 1

CB 62 LEU CB C 29.395 0.3 1

H 62 LEU H H 7.693 0.020 1

HA 62 LEU HA H 4.331 0.020 1

HB2 62 LEU HB2 H 1.985 0.020 1

HB3 62 LEU HB3 H 1.985 0.020 1

HG 62 LEU HG H 1.728 0.020 1

N 62 LEU N N 119.625 0.3 1

C 63 ALA C C 175.882 0.3 1

CA 63 ALA CA C 52.846 0.3 1

CB 63 ALA CB C 15.348 0.3 1

H 63 ALA H H 8.034 0.020 1

HA 63 ALA HA H 3.636 0.020 1

HB 63 ALA HB H 1.233 0.020 1

N 63 ALA N N 122.781 0.3 1

C 65 ARG C C 175.007 0.3 1

CA 65 ARG CA C 57.215 0.3 1

CB 65 ARG CB C 27.516 0.3 1

H 65 ARG H H 7.639 0.020 1

HA 65 ARG HA H 3.708 0.020 1

HB2 65 ARG HB2 H 1.874 0.020 1

HB3 65 ARG HB3 H 1.874 0.020 1

N 65 ARG N N 117.798 0.3 1

C 66 ASN C C 175.007 0.3 1

CA 66 ASN CA C 53.435 0.3 1

CB 66 ASN CB C 35.946 0.3 1

H 66 ASN H H 8.132 0.020 1

HA 66 ASN HA H 4.532 0.020 1

HB2 66 ASN HB2 H 2.880 0.020 1

HB3 66 ASN HB3 H 2.880 0.020 1

HD21 66 ASN HD21 H 7.094 0.020 1

N 66 ASN N N 117.005 0.3 1

C 67 ALA C C 176.184 0.3 1

CA 67 ALA CA C 52.533 0.3 1

CB 67 ALA CB C 15.362 0.3 1

H 67 ALA H H 8.345 0.020 1

HA 67 ALA HA H 4.660 0.020 1

HB 67 ALA HB H 1.435 0.020 1

N 67 ALA N N 123.376 0.3 1

C 68 ALA C C 176.891 0.3 1

CA 68 ALA CA C 52.223 0.3 1

CB 68 ALA CB C 15.664 0.3 1

H 68 ALA H H 8.568 0.020 1

HA 68 ALA HA H 4.058 0.020 1

HB 68 ALA HB H 1.527 0.020 1

N 68 ALA N N 119.272 0.3 1

C 69 ALA C C 178.438 0.3 1

CA 69 ALA CA C 52.524 0.3 1

CB 69 ALA CB C 15.365 0.3 1

H 69 ALA H H 8.514 0.020 1

HA 69 ALA HA H 3.966 0.020 1

HB 69 ALA HB H 1.527 0.020 1

N 69 ALA N N 119.440 0.3 1

C 71 MET C C 175.310 0.3 1

CA 71 MET CA C 51.659 0.3 1

CB 71 MET CB C 15.670 0.3 1

H 71 MET H H 8.387 0.020 1

HA 71 MET HA H 4.660 0.020 1

HB2 71 MET HB2 H 1.292 0.020 1

HB3 71 MET HB3 H 1.292 0.020 1

N 71 MET N N 120.698 0.3 1

CB 72 LEU CB C 29.395 0.3 1

H 72 LEU H H 8.751 0.020 1

HA 72 LEU HA H 3.709 0.020 1

N 72 LEU N N 121.565 0.3 1

H 73 LEU H H 6.998 0.020 1

HA 73 LEU HA H 4.001 0.020 1

HB2 73 LEU HB2 H 1.703 0.020 1

HB3 73 LEU HB3 H 1.703 0.020 1

HG 73 LEU HG H 1.543 0.020 1

N 73 LEU N N 116.181 0.3 1

C 74 ARG C C 176.004 0.3 1

CA 74 ARG CA C 54.952 0.3 1

CB 74 ARG CB C 26.605 0.3 1

H 74 ARG H H 7.504 0.020 1

HA 74 ARG HA H 3.865 0.020 1

HB2 74 ARG HB2 H 1.864 0.020 1

HB3 74 ARG HB3 H 1.864 0.020 1

N 74 ARG N N 119.653 0.3 1

C 75 VAL C C 174.368 0.3 1

CA 75 VAL CA C 61.297 0.3 1

CB 75 VAL CB C 28.785 0.3 1

H 75 VAL H H 8.172 0.020 1

HA 75 VAL HA H 3.764 0.020 1

HB 75 VAL HB H 2.058 0.020 1

HG1 75 VAL HG1 H 0.830 0.020 1

HG2 75 VAL HG2 H 0.830 0.020 1

N 75 VAL N N 113.698 0.3 1

C 77 THR C C 171.543 0.3 1

CA 77 THR CA C 59.712 0.3 1

CB 77 THR CB C 67.226 0.3 1

H 77 THR H H 7.614 0.020 1

HA 77 THR HA H 4.667 0.020 1

HB 77 THR HB H 4.219 0.020 1

HG2 77 THR HG2 H 1.160 0.020 1

N 77 THR N N 112.134 0.3 1

C 78 VAL C C 175.982 0.3 1

CA 78 VAL CA C 57.525 0.3 1

CB 78 VAL CB C 29.720 0.3 1

H 78 VAL H H 7.783 0.020 1

HA 78 VAL HA H 4.312 0.020 1

HB 78 VAL HB H 2.079 0.020 1

HG1 78 VAL HG1 H 0.976 0.020 1

HG2 78 VAL HG2 H 0.976 0.020 1

N 78 VAL N N 124.088 0.3 1

C 80 LEU C C 178.080 0.3 1

CA 80 LEU CA C 52.947 0.3 1

CB 80 LEU CB C 39.401 0.3 1

H 80 LEU H H 7.851 0.020 1

HA 80 LEU HA H 4.267 0.020 1

HB2 80 LEU HB2 H 2.538 0.020 1

HB3 80 LEU HB3 H 2.538 0.020 1

N 80 LEU N N 126.412 0.3 1

H 81 ASP H H 7.999 0.020 1

HA 81 ASP HA H 4.643 0.020 1

HB2 81 ASP HB2 H 2.895 0.020 1

HB3 81 ASP HB3 H 2.895 0.020 1

N 81 ASP N N 120.618 0.3 1

C 82 ALA C C 174.606 0.3 1

CA 82 ALA CA C 49.694 0.3 1

CB 82 ALA CB C 16.460 0.3 1

H 82 ALA H H 7.960 0.020 1

HA 82 ALA HA H 4.193 0.020 1

HB 82 ALA HB H 1.255 0.020 1

N 82 ALA N N 124.204 0.3 1

H 83 ARG H H 7.639 0.020 1

HA 83 ARG HA H 3.965 0.020 1

HB2 83 ARG HB2 H 1.603 0.020 1

HB3 83 ARG HB3 H 1.603 0.020 1

HE 83 ARG HE H 2.572 0.020 1

N 83 ARG N N 119.956 0.3 1

CB 84 ASP CB C 39.157 0.3 1

H 84 ASP H H 7.807 0.020 1

HA 84 ASP HA H 4.241 0.020 1

HB2 84 ASP HB2 H 2.517 0.020 1

HB3 84 ASP HB3 H 2.517 0.020 1

N 84 ASP N N 126.487 0.3 1

stop_
